# Parcellation‐based anatomic model of the semantic network

**DOI:** 10.1002/brb3.2065

**Published:** 2021-02-18

**Authors:** Camille K. Milton, Vukshitha Dhanaraj, Isabella M. Young, Hugh M. Taylor, Peter J. Nicholas, Robert G. Briggs, Michael Y. Bai, Rannulu D. Fonseka, Jorge Hormovas, Yueh‐Hsin Lin, Onur Tanglay, Andrew K. Conner, Chad A. Glenn, Charles Teo, Stéphane Doyen, Michael E. Sughrue

**Affiliations:** ^1^ Department of Neurosurgery University of Oklahoma Health Sciences Center Oklahoma City OK USA; ^2^ Department of Neurosurgery Prince of Wales Private Hospital Sydney NSW Australia; ^3^ Cingulum Health Sydney NSW Australia; ^4^ Omniscient Neurotechnology Sydney NSW Australia

**Keywords:** dual stream, language network, parcellation, tractography

## Abstract

**Introduction:**

The semantic network is an important mediator of language, enabling both speech production and the comprehension of multimodal stimuli. A major challenge in the field of neurosurgery is preventing semantic deficits. Multiple cortical areas have been linked to semantic processing, though knowledge of network connectivity has lacked anatomic specificity. Using attentional task‐based fMRI studies, we built a neuroanatomical model of this network.

**Methods:**

One hundred and fifty‐five task‐based fMRI studies related to categorization of visual words and objects, and auditory words and stories were used to generate an activation likelihood estimation (ALE). Cortical parcellations overlapping the ALE were used to construct a preliminary model of the semantic network based on the cortical parcellation scheme previously published under the Human Connectome Project. Deterministic fiber tractography was performed on 25 randomly chosen subjects from the Human Connectome Project, to determine the connectivity of the cortical parcellations comprising the network.

**Results:**

The ALE analysis demonstrated fourteen left hemisphere cortical regions to be a part of the semantic network: 44, 45, 55b, IFJa, 8C, p32pr, SFL, SCEF, 8BM, STSdp, STSvp, TE1p, PHT, and PBelt. These regions showed consistent interconnections between parcellations. Notably, the anterior temporal pole, a region often implicated in semantic function, was absent from our model.

**Conclusions:**

We describe a preliminary cortical model for the underlying structural connectivity of the semantic network. Future studies will further characterize the neurotractographic details of the semantic network in the context of medical application.

## Graphical abstract

Using 155 attentional task‐based fMRI studies, we built a neuroanatomical model of the semantic network described in the HCP parcellation scheme. Fourteen left hemisphere cortical regions were found to be part of the semantic network: 44, 45, 55b, IFJa, 8C, p32pr, SFL, SCEF, 8BM, STSdp, STSvp, TE1p, PHT, and PBelt. Notably, the anterior temporal pole, a region often implicated in semantic function, was absent from our model.

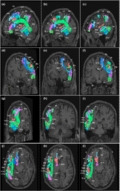

## 1 INTRODUCTION

The evolution of a complex semantic network, one that enables us to create language and encode meaning, has fascinated researchers since the origins of neuroscience, and is considered to be a defining characteristic of human beings. Our experience helps mold and build “knowledge” of all objects in the environment, which is reflected through language, and is pivotal for the comprehension of word meanings, with the subsequent retrieval of this stored knowledge known as “Semantic processing.” In addition to its fundamental role in language, semantic processing is highly influential for higher‐order cognitive processes including problem‐solving, planning and reasoning, emphasizing the integral role it plays in executive function (Binder et al., 2009; Tomasello et al., 2018).

Being the focus of studies for decades, locating and ascertaining the function of prospective cortical loci has been contentious, with neuroimaging and neurophysiological studies directed toward the existence of cortical regions, ranging from “semantic hubs” associated with semantic processing, to sensorimotor areas, that are modality preferential areas involved in processing abstract information (Tomasello et al., 2018; Xu et al., 2017). The semantic network model has continued to evolve since the pioneering Broca‐Wernicke‐Lichtheim‐Geschwind “classical model,” that implicated the temporal, frontal, and parietal lobes in semantic processing (Chang et al., 2015; Tremblay & Dick, 2016). A significant contemporary leap was made through the introduction of Hickok and Poeppel's neuroanatomical “Dual Stream Model,” which accentuated dorsal and ventral pathway for processing in concordance with visual processing theories (Hickok & Poeppel, 2007; Saur et al., 2010). This model posits that speech sound processing is initiated in the posterior superior temporal gyrus (STG) and superior temporal sulcus (STS). From this area, the ventral semantic stream flows through the anterior and middle temporal lobe, while the dorsal semantic stream flows through the parieto‐temporal boundary to the frontal lobe (Chang et al., 2015). The dual‐stream model is supported by insights from lesion studies, intraoperative cortical mapping, and subcortical fiber mapping (Chang et al., 2015). While there is some agreement on the regions of the cortex comprising the semantic network, such as the superior temporal sulcus and inferior frontal gyrus (Chang et al., 2015; Duffau et al., 2014), existing descriptions of the semantic network lack anatomic specificity and offer limited insight into the underlying structural connectivity of the network. Additionally, without the use of unanimous nomenclature to describe this network, it prevents comparison between studies.

In this study, we constructed a model of the semantic network based on the cortical parcellation scheme published from the Human Connectome Project (HCP; Glasser et al., 2016). The creation of the “multi‐modal parcellation” of the labyrinth like cerebrum through functional magnetic resonance imaging (fMRI) helped provided an accurate map of the cortical and subcortical areas, which facilitates the comparison of data and provides an anatomical structure to base studies on. Using relevant task‐based fMRI, PubMed, and BrainMap (http://www.brainmap.org/), a collection of open‐access software programs used to generate activation likelihood estimations from fMRI data, we identified the primary cortical areas involved in the semantic network. After identifying these regions of interest, we performed 637 tractography to determine the structural connectivity between parcellations of the network.

## 2 METHODS

### 2.1 Literature search

We searched Brainmap Sleuth 2.4 (Fox et al., 2005; Fox & Lancaster, 2002; Laird et al., 2005; Vanasse et al., 2018) on 24 July 2017 for all relevant task‐based fMRI studies related to semantic processing in healthy individuals. The following search algorithm was used: “A. Experiments: Imaging Modality = fMRI, B. Experiments: Behavioral Domain = Cognition: Language, C. Experiments: Paradigm Class = Semantic Monitor/Discrimination, D. Subjects: Diagnosis = Normals, E. Conditions: Stimulus = Auditory Words/Stories, Visual Words, and Visual Images.” The Semantic Monitor/Discrimination Paradigm Class includes experiments that require individuals to discriminate between the meanings of select lexical items or to indicate if a target word is semantically related to a probe word. The stimuli presented in these discrimination tasks can be auditory words, visual words, or pictures representing words. The fifth search criterion (E. Conditions: Stimulus) was varied to ensure inclusion of studies related to all three types of task‐based stimuli.

**FIGURE 1:**
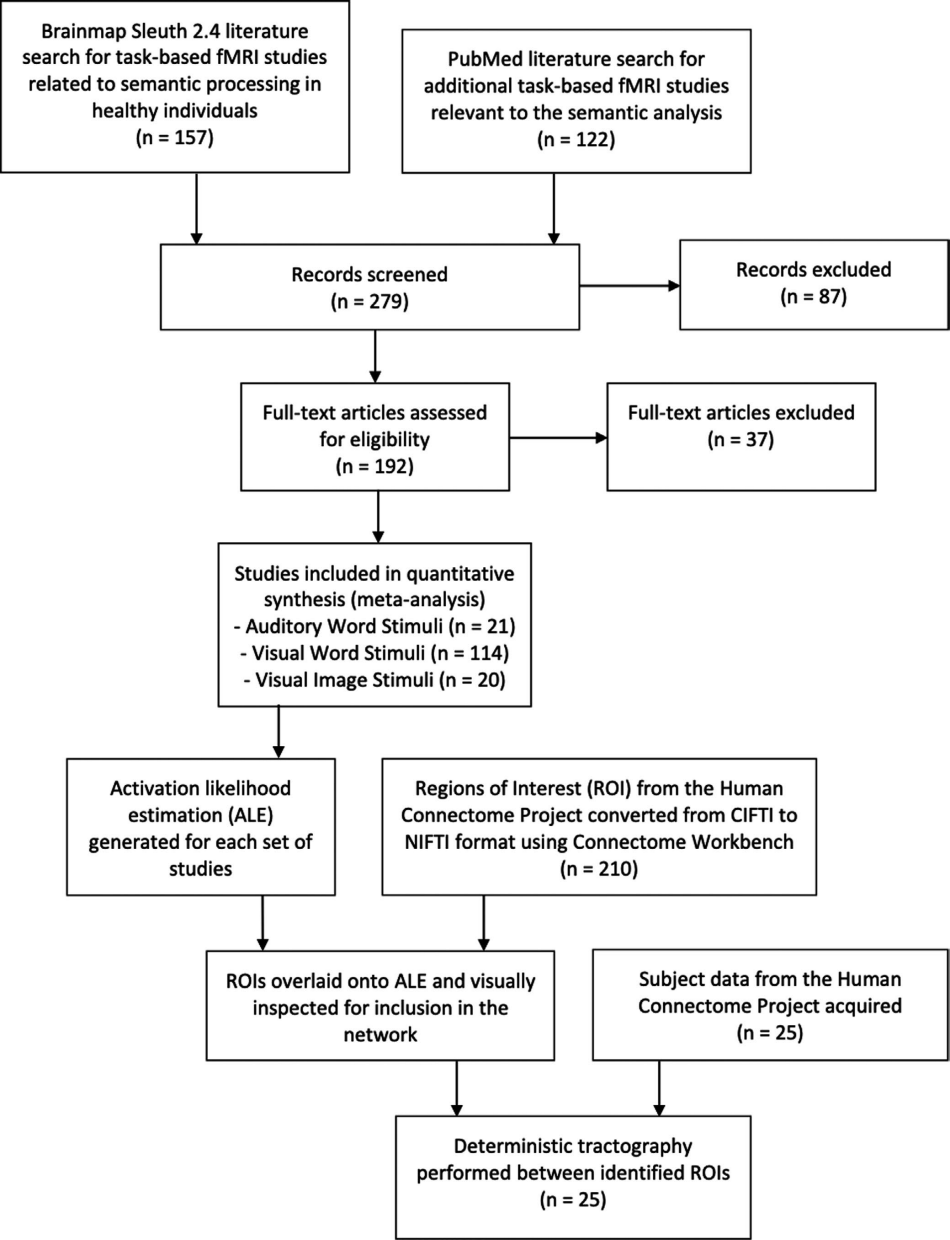
Flow diagram demonstrating the methods used in this study

PubMed was utilized to acquire studies relevant to the semantic network and included in our analysis if they met the following criteria (1) peer‐reviewed publication, (2) task‐based fMRI study related to the semantic processing (Fox et al., 2005;Fox et al., 2002; Laird et al., 2005), based on whole‐brain, voxel‐wise imaging, (4) including standardized coordinate‐based results in the Talairach or Montreal Neuroimaging Institute (MNI) coordinate space, and (5) including at least one healthy human control cohort. Only coordinates from healthy subjects were utilized in our analysis. Region of interest studies, meta‐analyses, resting‐state studies, and studies examining interactions between two or more networks were excluded. Overall, 21 papers related to auditory word stimuli, 114 papers related to visual word stimuli, and 20 papers related to visual image stimuli met criteria for inclusion in this study. The details of these studies are summarized in Tables S1–S3, and a flow chart of our methods is seen in Figure 1. It is important to note that studies were not excluded based on the hemispheric activation (i.e., right‐sided activation only), any studies that meant the above criteria were included in the analysis.

### 2.2 Creation of 3D regions of interest

The parcellation data acquired from the HCP study was analyzed using surface‐based greyordinates, through a CIFTI file format, which helped generate regions of interest (ROI’s; Glasser et al., 2016). As it was challenging to carry out tractography using surface‐based greyordinates, they had to be converted into volumetric coordinates, which involves traditional formats including NIFTI. This process was completed using the CIFTI separate and ‐label‐resample Workbench Commands in the Connectome Workbench (Van Essen Laboratory, Washington University, 2016; Glasser et al., 2013) and the Freesurfer commands “mris_convert” and “mri_annotation2label” (Fischl, 2012) that used previously published structural imaging data from HCP authors, and standardized the greyordinate label parcellations to three‐dimensional volumetric working spaces of DSI studio (http://dsi‐studio.labsolver.org). The authors were able to perform deterministic fiber tractography on 180 left hemispheric parcellations that had been converted to volumetric coordinates.

### 2.3 ALE generation and identification of relevant cortical regions

We used BrainMap Ginger ALE 2.3.6 to extract the relevant fMRI data for creation of an activation likelihood estimation (ALE) related to each set of papers for a given stimulus category (Eickhoff et al., 2009, 2012; Turkeltaub et al., 2012). All coordinates were exported to Ginger ALE in the Talairach coordinate space. We subsequently performed a Single Study analysis using Cluster‐Level Inference (cluster level of .05, threshold permutations of 1,000, uncorrected *p*‐value of .001). The ALE coordinate data were displayed on a Talairach‐normalized Colin‐27 template brain using the Multi‐image Analysis GUI (Mango) 4.0.1 (ric.uthscsa.edu/mango). The preconstructed ROIs of the parcellations were then overlaid on the ALE and compared visually for inclusion in the network. Following this, the preconstructed ROI parcellation data were compared with the ALE cluster analysis data. The cluster analysis is an output from Ginger ALE and reveals the centroid and size of each ALE cluster. An analysis was run, wherein these clusters were compared to the ROI parcellation data, and a list of parcellations that fell within each cluster was created. The percentage that each parcellation was within any one cluster was created and analyzed, and a cut‐off value of 15% was used to determine inclusion in the semantic network.

### 2.4 Tractography

Working from the hypothesis that functionally connected regions of a network are likely structurally connected, we proceeded to determine the backbone of the network using deterministic tractography. Deterministic tractography was chosen as the method of tractography, rather than probabilistic methods, as they have been more successfully incorporated into clinical practice, such as for neurosurgical planning, and thus we wanted the findings of this study to be clinically applicable. All fiber tractography was completed in DSI Studio (http://dsi‐studio.labsolver.org) using publicly available brain imaging from the HCP (http://humanconnectome.org, release Q3). Tractography was performed individually with 25 randomly chosen healthy adult subjects (Subjects IDs: 100307, 103414, 105115, 110411, 111312, 113619, 115320, 117112, 118730, 118932, 100408, 115320, 116524, 118730, 123925, 148335, 148840, 151526, 160123, 178950, 188347, 192540, 212318, 366446, 756055).

A multi‐shell diffusion scheme was used, with *b*‐values of 990, 1,985, and 1,980 s/mm^2^. Each *b*‐value was sampled in 90 directions. The in‐plane resolution was 1.25 mm. The slice thickness was 1.25 mm. The diffusion data were reconstructed using generalized q‐sampling imaging (Yeh et al., 2010). The diffusion sampling length ratio was 1.25.

All reconstructions were performed in MNI space using a ROI approach to initiate fiber tracking from a seeded region. Greyordinate label parcellation fields were standardized to the three‐dimensional volumetric working spaces of DSI studio using the structural imaging data provided by HCP for each subject. Voxels within each ROI were automatically traced with a maximum angular threshold of 45°. When a voxel was approached with no tract direction or a direction greater than 45°, the tract was halted. Tracks with length shorter than 30 mm or longer than 300 mm were discarded. In some instances, exclusion ROIs were placed to exclude spurious tracts or tracts inconsistently represented across individuals. Tracts were considered meaningful between parcellations if they could be identified consistently in five or more subjects.

### 2.5 Ethical statement

This study does not require an ethical statement as no human or animal subjects were used.

## 3 RESULTS

### 3.1 ALE regions and corresponding parcellations

**FIGURE 2:**
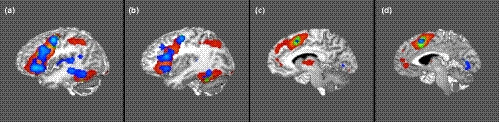
Activation Likelihood Estimation (ALE) of 155 task‐based fMRI experiments related to goal‐oriented attentional processing, wherein red data represents the ALE of the visual word stimuli studies, blue represents the auditory words and stories studies, and green is the ALE data of visual image stimuli studies. The three‐dimensional ALE data are displayed in Mango on a brain normalized to the MNI coordinate space. ALE data highlighting the left lateral occipital lobe. (a–d) ALE data highlighting the left superior parietal lobule and intraparietal sulcus. (c and d) ALE data highlighting the left frontal eye field of the frontal lobe

Figure 2 demonstrates the ALE of the 155 task‐based fMRI experiments included in our meta‐analysis. The highlighted areas within this ALE were each named based on their location (Lateral Frontal [LF], Medial Frontal [MF], Parietal [P], Posterior Temporal [T], or Primary Auditory Cortex [AC]) and associated stimulus (AWS, VW, or VI). Thirteen distinct left hemispheric regions of the cortex were highlighted in the ALE: four regions associated with the lateral frontal lobe (LF‐AWS1, LF‐AWS2, LF‐VW, LF‐VI); three associated with the medial frontal lobe (MF‐AWS, MF‐VW, MF‐VI); four associated with the posterior temporal lobe (T‐AWS, T‐VW, T‐VI1, T‐VI2); one parietal lobe region (P‐VW); and one auditory cortex region (AC‐AWS). The ALE data are summarized in Figure 2.

**FIGURE 3:**
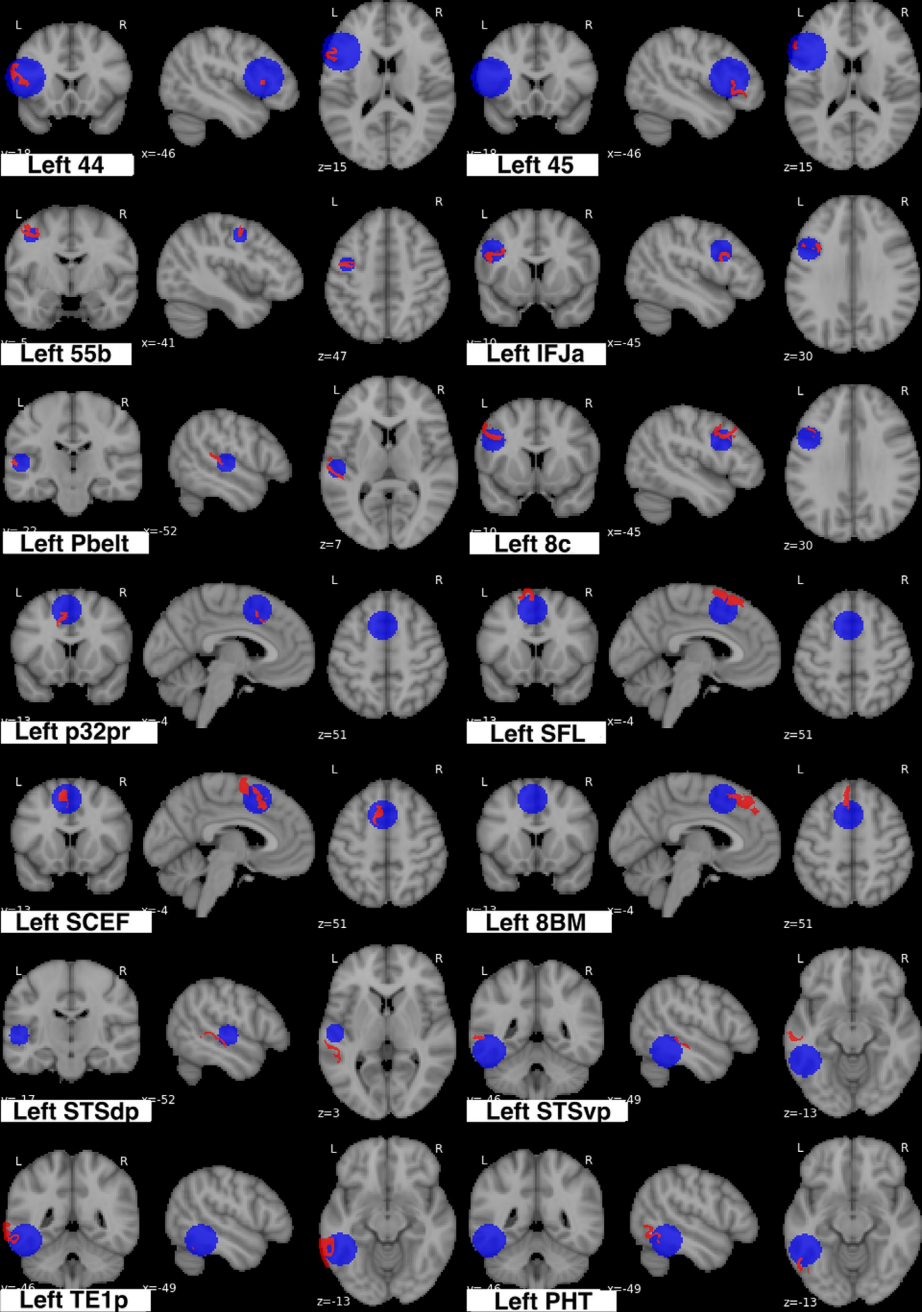
Comparison overlays between the cortical parcellation data (red) and activation likelihood estimation (ALE) cluster data (blue) of the semantic network. Regions were visually assessed for inclusion in the network if they overlapped with the ALE data. To confirm these findings, we underwent an analysis of how much each parcellation overlapped with the ALE clusters, which were provided as an output of the ALE data. Any parcellation that fell more than 15% within the ALE cluster was included in the network

Integrating the parcellations data, fourteen left hemispheric regions of interest overlapped with the ALE: 8C, 44, 45, 55b, and IFJa of the lateral frontal lobe; 8BM, p32pr, SCEF, and SFL of the medial frontal lobe; PBelt of the primary auditory cortex; STSdp, STSvp, PHT, and TE1p of the posterior lateral temporal lobe. The visual assessment included left hemispheric areas AIP and PFm into the network, however, they fell below the 15% cut‐off in the cluster analysis, and therefore were excluded from our model of the network. Comparison overlays between the cortical parcellations and the ALE are shown in Figure 3 and Table 1 represents the percentage of each parcellation that falls within the ALE.

**TABLE 1:** Percentage of each parcellation that falls within the Activation Likelihood Estimation (ALE) clusters

Parcellation Name	Percentage of parcellation within ALE
L_44	100.00
L_45	68.21
L_55b	82.15
L_IFJa	97.43
L_8C	55.74
L_p32pr	59.74
L_SFL	26.60
L_SCEF	73.06
L_8BM	15.93
L_STSdp	20.35
L_STSvp	16.00
L_TE1p	39.53
L_PHT	23.88
L_PBelt	45.76

### 3.2 Structural connectivity of the semantic network

**FIGURE 4:**
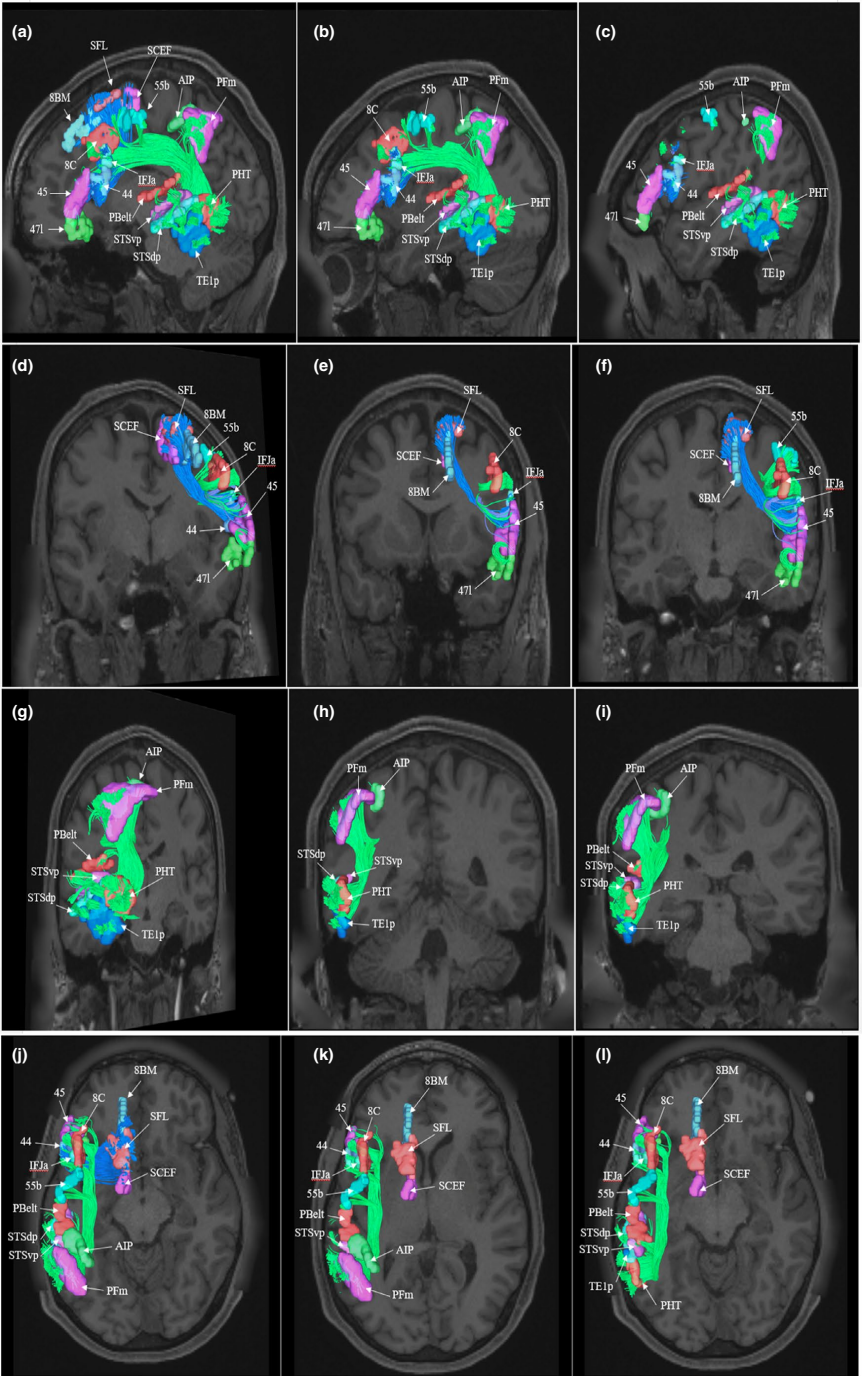
Fiber tracking analysis for the semantic network. Shown on T1‐weighted MR images in the left cerebral hemisphere. TOP ROW: sagittal sections from most medial to most lateral demonstrating the superior longitudinal fasciculus and its projections between the frontal, parietal, and temporal clusters of the dorsal attention network. ROW TWO AND THREE: Partially oblique (left column) and pure (middle and right column) coronal sections. BOTTOM ROW: axial sections through the frontal and parietal clusters of the network. The fronto‐parietal projections of the SLF are particularly apparent

**FIGURE 5:**
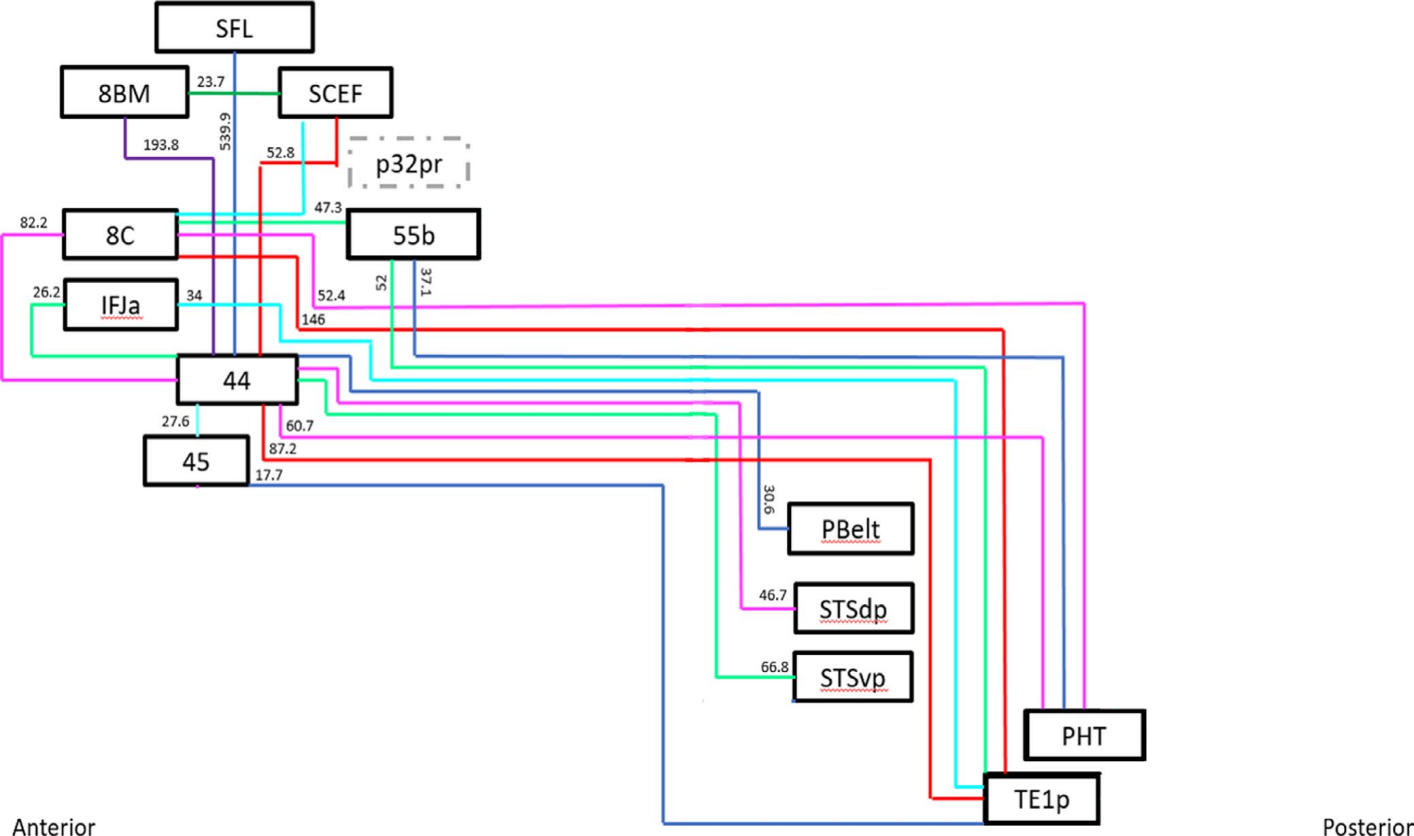
Simplified schematic of the white matter connections identified between individual parcellations of the semantic network during the fiber tracking analysis. Connections are labeled with the average strength measured across all 25 subjects

Deterministic tractography was utilized to determine the basic structural connectivity of the semantic network. These results are shown in Figure 4. The individual connections within the network are presented in Table 2, which presents the strength of individual connections and mentions the type of white matter connections networking the regions. ROIs showed consistent local connections between adjacent parcellation that were observed consistently are summarized in Figure 5.

**TABLE 2:** Type and strength of connections within the semantic language network

Connection	Number of subjects	Average strength weighted by all subjects	Average strength weighted by identified subjects	Connection type
SFL to 44	25/25 (100%)	539.9	539.9	FAT
8BM to 44	23/25 (92%)	193.8	210.7	FAT
8BM to SCEF	8/25 (32%)	23.7	74.1	U‐shaped Fiber
SCEF to 44	13/25 (52%)	52.8	101.5	FAT
8C to TE1p	11/25 (44%)	146	332	SLF
8C to 44	21/25 (84%)	82.2	100.3	U‐shaped Fiber
8C to 55b	12/25 (48%)	47.3	98.5	U‐shaped Fiber
8C to PHT	5/25 (20%)	52.4	262	SLF
IFJa to 44	13/25 (52%)	26.2	50.5	U‐shaped Fiber
IFJa to TE1p	8/25 (32%)	34	106.4	SLF
44 to TE1p	16/25 (64%)	87.2	163.3	SLF
44 to PHT	6/25 (24%)	60.7	252.8	SLF
44 to STSvp	14/25 (56%)	66.8	119.4	SLF
44 to STSdp	13/25 (52%)	46.7	89.8	SLF
44 to Pbelt	11/25 (44%)	30.6	69.5	SLF
44 to 45	14/25 (56%)	27.6	49.3	U‐shaped Fiber
45 to TE1p	4/25 (16%)	17.7	110.8	SLF
45 to 47l	15/25 (60%)	51.3	85.5	U‐shaped Fiber
55b to PHT	11/25 (44%)	37.1	84.3	SLF
55b to TE1p	6/25 (24%)	52	216.8	SLF
PHT to TE1p	13/25 (52%)	44.9	86.4	U‐shaped Fiber

The cortical regions included in our model of the semantic network can be categorized into two general clusters based on anatomical specification: a left frontal cluster (8C, 44, 45, 55b, IFJa, SCEF, and SFL) and a left temporal cluster (PBelt, STSdp, STSvp, PHT, and TE1p). The superior longitudinal fasciculus (SLF) represented the majority of the connections between ROI pairs of the network (16/27 connections, 59%). The SLF projects between the frontal, parietal, and temporal clusters as it courses within the subcortical white matter around the Sylvian fissure (see Figure 4). In general, connections of the SLF within the semantic network can be classified into three subtypes: fronto‐parietal, parieto‐temporal, and fronto‐temporal.

The U‐shaped fibers were also found to connect cortical areas within the Semantic Network (8/27 connections, 29.6%). These fibers generally have the same morphology, arising within one part of the cortex before curving 180 degrees to terminate in a part of the brain immediately adjacent to its origin. The U‐shaped fibers represent the local connections between the frontal, parietal, and temporal zones.

## 4 DISCUSSION

In this study, we utilized meta‐analytic software and deterministic tractography to construct a model of structural connectivity of the semantic network based on the cortical parcellation scheme published previously under the HCP (Glasser et al., 2016). This would provide us with a critical framework of the cortical location of the semantic function, and the associated interconnected areas, that would behave as a basis for future validation studies. We believe that a better understanding of the cortical areas and tracts involved in the semantic network would play a pivotal role in improving neurosurgeons’ ability to preserve this network's function in surgery.

### 4.1 The dual‐stream model of semantic function

Neurological models of semantic organization have generated controversy in all of their historical iterations. The Wernicke‐Geschwind model of language function which was developed in the late 1800s described a single white matter tract connecting the anterior inferior frontal lobe (Broca's Area) with the posterior temporal lobe (Wernicke's Area; Tremblay & Dick, 2016). While this model has been largely abandoned, this nomenclature persists. The varying anatomical definitions of Broca's Area and Wernicke's Area has contributed to confusion between the modern dual‐stream models of language processing (Tremblay & Dick, 2016).

The modern Hickok‐Poeppel and Rauschecker‐Scott dual‐stream models theorize that the dorsal stream functions in sensorimotor integration of language (Hickok & Poeppel, 2004, 2007; Rauschecker & Scott, 2009), while the ventral stream is responsible for recognition of speech as well as conceptual representations of language (Chang et al., 2015). A major source of contention between various semantic models is the role of the ventral stream in the dual‐stream model. It has been suggested that lesions to a ventral tract connecting the frontal and posterior temporal lobes results in semantic paraphasias, with both the IFOF and UF being proposed. (Chang et al., 2015; Duffau et al., 2005; Duffau et al., 2014). Nevertheless, understanding of the ventral stream is complicated by inconsistent identification of the anterior temporal lobe fMRI imaging studies, specifically the failure of fMRI studies to report activation of the anterior temporal lobe during semantic tasks, with emphasis directed toward prefrontal and temporo‐parietal regions instead (Binder et al., 2011; Visser et al., 2010).

### 4.2 Critical re‐evaluation of the semantic language network

This study does not aim to create an exhaustive model of the Semantic Network. Our Sleuth literature search was limited to functional studies that required patients to recognize conceptual categories of auditory words, written words, and visual representations of words. However, we argue that the ability to place multimodal stimuli into conceptual categories constitutes fundamental semantic function. Notably, regions identified as part of the anterior temporal ventral semantic stream are absent from our model. We hypothesize, therefore, that this specific categorization task paradigm does not involve the ventral pathway to a significant extent. An important aspect to note is that our ALE generated solely left hemispheric regions. With some exceptions, most models of the semantic dual‐stream state that while language is a strongly left‐lateralized function, the right side of the brain may also play an important role in certain semantic processes (Boemio et al., 2005; Chang et al., 2015; Duffau et al., 2014). Despite these caveats, our proposed semantic model appears to coincide with many aspects of the contemporary Dual‐Stream Semantic Models presented by the literature, especially the frameworks proposed by Hickok & Poeppel in 2004 and elaborated on by Duffau et al. in 2014 (Duffau et al., 2014; Hickok & Poeppel, 2004).

### 4.3 Putative functions of relevant cortical regions

In order to move toward an anatomical model of the dorsal stream, it is critical that we understand the specific subset of semantic function served by each of the regions we identified in our network. Many of the HCP parcellations are newly identified regions or subregions of previously identified areas. Therefore, the specific function of all of these areas is not fully understood. Nevertheless, the function of the larger cortical regions in which the parcellations are located has been hypothesized. For the sake of clarity, we have grouped the 18 parcellations into five categories based on anatomic location.

### 4.4 Superior temporal sulcus and the supramarginal gyrus

The STS has been consistently implicated in the processing of phonological information (Chang et al., 2015; Duffau et al., 2014; Hickok & Poeppel, 2004, 2007). The posterior STS and STG, where the parcellation STSdp and STSvp are located, may represent the contemporary Wernicke's area as it is involved in spectro‐temporal and phonological analysis (Chang et al., 2015). PBelt is a newly identified region recognized as part of the early auditory cortex (Glasser et al., 2016). It is located in the space between the lateral edge of Heschl's gyrus and the opercular cleft of the inferior SMG (Glasser et al., 2016).

### 4.5 Inferior frontal gyrus and premotor cortex

Parcellations 44 and 45 were named for their significant overlap with Brodmann Areas 44 and 45, the traditional Broca's Area (Glasser et al., 2016). 47L is a neighboring parcellation that Glasser and colleagues named for its overlap with regions described in previous studies (Van Essen et al., 2012). Broca conceptualized this region as housing the brain's “motor‐word image” necessary for language articulation (Chang et al., 2015).The Inferior Frontal Gyrus is no longer considered the only important destination for the dorsal stream. But it is still thought to represent a critical destination of the dorsal stream, responsible for “mapping phonological information onto articulatory‐motor representations” of language (Chang et al., 2015).

In conjunction with Broca's Area, more dorsal areas of the frontal lobe are hypothesized to function in speech articulation. In addition, phonological working memory tasks activate “fronto‐parietal loop” linking the dorsal inferior frontal gyrus with the dorsal supramarginal gyrus (Vigneau et al., 2006). Duffau et al. (2014) proposed that this working memory loop provides temporary storage of the phonemes making up a word or sentence. Area 55b was identified in the HCP parcellation scheme for its distinctive functional connectivity to other distant parcellations activated by language tasks (Glasser et al., 2016).

### 4.6 Medial prefrontal cortex

SFL, or Superior Frontal Language Area, is a new parcellation in the dorsolateral prefrontal cortex identified by the HCP that showed strong left lateralization and strong activation in language tasks (Glasser et al., 2016). a32pr and p32pr are newly described cortical parcellations identified by the HCP that overlap with area 32 in Vogt's parcellation (Glasser et al., 2016; Vogt et al., 1995).

### 4.7 Middle temporal gyrus

Parcellations TE1p and PHT lie in the posterior middle temporal gyrus (MTG). The Hickock‐Poeppel model of the semantic network posits that the left posterior inferior temporal lobe (PITL), containing posterior sections of both the MTG and inferior temporal gyrus, serves to integrate auditory representations of language from the STG with multimodal conceptual representations of language (Hickok & Poeppel, 2004, 2007). Indeed, the posterior MTG has been shown to be important for accessing lexical and semantic information (Hickok & Poeppel, 2007). Modifying the Hickock‐Poeppel model's description of the PITL as a sound‐meaning interface, Duffau et al. (2014) suggested that this region also functions in picture naming as a “Visual Object Form Area” which receives input from occipital lobe visual areas. Furthermore, Duffau's model implicates this region as a common hub between the dorsal syntactic stream and the ventral semantic stream (Duffau et al., 2014).

### 4.8 Subcortical connections within the semantic network

Language is one of the most widely distributed functional networks in the brain. The evolution of long‐range white matter connections between auditory, visual, and motor areas illustrates the multimodal complexity of semantic processing (Turken & Dronkers, 2011). While the arcuate fasciculus (AF) has long been thought to be the critical white matter pathway underlying semantic function, the list of potentially language‐related tracts has broadened. Our tentative model supports the role for the recently identified frontal aslant tract in semantic function (Chang et al., 2015; Turken & Dronkers, 2011).

Four major subdivisions of the SLF have been described previously wherein three have been implicated in semantic processing (Chang et al., 2015). SLF II links the dorsal premotor and prefrontal areas and the angular gyrus, SLF III connects the frontal operculum with the supramarginal gyrus, and SLF‐tp runs vertically between the inferior parietal lobe and the posterior temporal lobe (Chang et al., 2015). Stimulation of both the superior SLF II and the inferior SLF III components have been shown to elicit speech arrest and dysarthria, while stimulation of SLF III has been shown to cause repetition errors (Chang et al., 2015). These language deficits support the hypothesis of an articulatory processing function for the SLF II and SLF III components (Chang et al., 2015). The AF, which may be considered a deep component of the SLF, was historically thought to connect Broca's and Wernicke's Areas. Stimulation of both SLF‐tp and the AF elicits phonological paraphasias, supporting a phonological processing function for these tracts (Chang et al., 2015).

Based on our current functional and anatomical understanding of the white matter tracts spanning our cortical semantic regions, we can form several hypotheses to support our dorsal stream model. (1) The white matter tracts connecting the “Inferior Frontal Gyrus and Premotor Cortex” parcellations with the “Inferior Parietal Lobule” parcellations represent SLF II and SLF III and serve an articulatory function. (2) The white matter tracts connecting the “Inferior Parietal Lobule” parcellations to the “Posterior MTG” areas represent SLF‐tp which serve a phonological processing function (3) The white matter tracts connecting Broca's area (44) with STSdp represents the AF (deep part of the SLF) which functions primarily in phonological processing. (4) The white matter tracts connecting the “Inferior Frontal Gyrus and Premotor Cortex” areas with the “Medial Prefrontal Cortex” areas are the frontal aslant tract, which has an unknown role in semantic processing.

### 4.9 Significant findings regarding role of the anterior temporal lobe in semantic function

Proponents of the anterior temporal lobe's role in semantic processing have implicated it as an “amodal hub” responsible for the integration and retrieval of conceptual semantic information from auditory and visual areas (Binder et al., 2011; Visser et al., 2010), a function performed in all three task paradigms of our meta‐analysis in the absence of anterior temporal lobe activation. We propose, therefore, that regions typically identified as belonging to the dorsal stream do not serve an exclusively phonological or syntactic function as proposed by other models (Chang et al., 2015; Duffau et al., 2014; Hickok & Poeppel, 2004) but are also responsible for the undeniably *semantic* function of recognizing meaning.

Our functional meta‐analysis of multimodal word categorization tasks does not show activation of anterior temporal regions that theoretically belong to the ventral stream (Duffau et al., 2014; Hickok & Poeppel, 2004, 2007). By specifically defining *semantic function* as activities requiring conceptual categorization of language, we have failed to demonstrate a role for the anterior temporal lobe. Thus, we argue that the anterior temporal lobe cortical and subcortical regions commonly identified as part of the ventral semantic stream may not serve a truly *semantic* *function*. We do not intend to deny the role of the anterior temporal lobe in language‐related activities but propose the modification of terminology used to describe its function.

### 4.10 Limitations

It is important to recognize that the deterministic tractography used in the present study cannot account for inherent uncertainties in estimates of fiber orientations and it can be susceptible to noise, when compared to probabilistic tractography (Maier‐Hein et al., 2017). Nonetheless, we use deterministic tractography as it has been more successfully incorporated into clinical practice, which is the goal of this study, and it has been demonstrated to outperform probabilistic methods in tractography algorithms, particularly in human connectome mapping (Maier‐Hein et al., 2017; Sarwar et al., 2019). Additionally, while the present study did not suggest a role of the anterior temporal lobe within the semantic network, this could be due to the inherent limitations of fMRI signal's being compromised by artifacts and signal distortions at this region due to its proximity to nasal sinuses and ear canals (Devlin et al., 2000). Additionally, some fMRI studies use a restricted field of view which can exclude the inferior anterior temporal lobe from the acquired images (Visser et al., 2010). Further, while the nature of meta‐analyses has its benefits in that it mitigates the single‐center effect and increases statistical power to emphasize similarities between studies, it is inherently limited by the input data. This is furthered by the heterogeneity of imaging data, especially in that our study includes data spanning many years. While we have attempted to mitigate this heterogeneity with our selection criteria, it is impossible to completely eliminate this to a set of experiments.

## 5 CONCLUSIONS

We present a preliminary tractographic model of the semantic language network. Based on the results of a literature meta‐analysis, we have provided evidence that the semantic categorization of visual and auditory stimuli activates a widespread dorsal network of cortical regions, corresponding to 14 regions from the HCP parcellation scheme. We have identified regions 44 and 55b as dorsal semantic network “hubs” based on their high level of connectivity to both local and distant semantic regions. For the purpose of clinical translation, we believe studies like this can be used as prior knowledge to interpret resting‐state fMRI, which is often easier to obtain in a clinical setting. Further studies may refine this model with the ultimate goal of clinical application.

## DISCLOSURE

Dr. Sughrue is the Chief Medical Officer and Stephane Doyen is the Chief Data Scientist and Technology Offer of Omniscient Neurotechnology. Hugh Taylor and Peter Nicholas are employees of Omniscient Neurotechnology. No products related to this were discussed in this paper. The other authors report no conflicts of interest.

## AUTHOR CONTRIBUTIONS

**Camille K. Milton:** Investigation, Validation, Writing—Original Draft. **Vukshitha Dhanaraj:** Investigation, Formal Analysis. **Isabella M. Young:** Formal Analysis, Writing—Review & Editing, Revisions. **Hugh M. Taylor:** Software, Visualization, Revisions. **Peter J. Nicholas**: Methodology, Software, Revisions. **Robert G. Briggs:** Writing—Review & Editing. **Michael Y. Bai**: Visualization. **R. Dineth Fonseka**: Visualization, Data Curation^.^ **Jorge Hormovas:** Investigation, Revisions. **Yueh‐Hsin Lin:** Data Curation. **Onur Tanglay:** Resources. **Andrew K. Conner:** Data Curation. **Chad A. Glenn:** Project Administration. **Charles Teo:** Supervision, Revisions. **Stéphane Doyen:** Supervision, Software, Revisions. **Michael E. Sughrue:** Conceptualization, Supervision.

### PEER REVIEW

The peer review history for this article is available at https://publons.com/publon/10.1002/brb3.2065.

## Supporting information

Table S1

Table S2

Table S3

## Data Availability

The data that support the findings of this study are available from the corresponding author (MS), upon reasonable request.
