# Supplementary material for: Parcellation‐based anatomic model of the semantic network
Source: Brain Behav. 2021 Feb 18;11(4):e02065. doi: 10.1002/brb3.2065 (PMC8035438; doi:10.1002/brb3.2065)
Supplement: Supplementary file 1 — Table S1 [file BRB3-11-e02065-s003.docx]

Table S1. Studies Related to the Auditory Words and Stories Task Paradigm

| **Brain Map ID** | **Year** | **First Author** | **Journal** | **Subjects** | **Experiment Number** | **Experiment Name** | **Coordinate**  **Space** | **Coordinates** |
| --- | --- | --- | --- | --- | --- | --- | --- | --- |
| 30006 | 1996 | Binder J R | Brain | 12 | 2 | Words > Tones - Active | Talairach | -45 32 3  -53 -12 -10  -46 15 30  -57 -34 -11  -45 -74 29  -45 -35 -11 |
| 30079 | 2002 | Booth J R | Human Brain Mapping | 13 | 3 | Auditory Meaning - Control | MNI | -48 18 18  -12 0 15  -9 -78 6  -51 -18 -6  63 -15 -6  33 -21 -9  -6 -33 -9  -45 36 -15  12 -81 -27 |
| 30079 | 2002 | Booth J R | Human Brain Mapping | 13 | 7 | Auditory Meaning - Rhyming | MNI | -51 21 18  -21 -93 3  -45 -33 -6  24 -72 -12  12 -81 -30 |
| 30079 | 2002 | Booth J R | Human Brain Mapping | 13 | 9 | Meaning - Rhyming | MNI | -6 18 42  -54 21 18  48 30 18  12 -81 12  -27 -63 0  18 -84 -3  -48 -33 -6  -48 21 -9 |
| 16030057 | 1999 | Chee M W | Human Brain Mapping | 8 | 3 | Auditory Abstract/Concrete > Fixation | Talairach | 40 48 6  25 57 6  -28 57 0  43 39 21  34 45 34  25 6 21  -31 -6 0  37 18 6  -31 18 12  -46 27 25  -43 3 28  -40 -6 46  37 -24 59  9 3 53  3 -9 59  -21 -9 65  34 0 68  59 -18 9  62 -33 12  -59 -18 15  -37 -54 -9  37 -33 65  -46 -42 53  -43 -54 53  3 -54 -15  34 -48 -31  9 -66 -25  -25 -51 -21  0 -27 -31  -6 0 21 |
| 16030057 | 1999 | Chee M W | Human Brain Mapping | 8 | 5 | Abstract/Concrete > Syllable | Talairach | 31 54 3  53 42 6  -43 42 0  -40 36 15  34 18 -3  -28 18 15  -37 15 -3  50 36 -6  -46 30 0  -46 18 34  -46 9 21  -40 -6 46  -6 9 65  -6 24 43  -28 6 50  -15 3 34  -56 -36 0  -59 -6 -15  -37 -54 -9  28 -57 -37  15 -30 -28  15 -69 -34  -34 -45 -21  -25 -60 -31  -18 -30 -31 |
| 16030057 | 1999 | Chee M W | Human Brain Mapping | 8 | 6 | Abstarct/Concrete > Case | Talairach | 31 51 3  -34 48 3  -31 21 15  -43 33 9  -40 15 31  -37 27 0  -25 33 0  40 -6 56  -3 9 62  12 -63 -37 |
| 30432 | 1999 | Curtis V A | Schizophrenia Research | 5 | 4 | Group x Task Interactions | Talairach | -17 -81 -7  20 -78 4  0 -47 48  6 -58 20  14 -86 9  -46 6 42  -46 14 4  -29 -72 -13  32 -58 -13  -38 -53 -13  3 6 53  26 -81 20 |
| 30276 | 1999 | Dapretto M | Neuron | 8 | 1 | Syntactic vs. Rest | MNI | -52 10 28  -40 30 14  -58 -58 14  -60 -36 16  -48 20 -16  56 10 -10  -42 -56 38  40 -50 42 |
| 30276 | 1999 | Dapretto M | Neuron | 8 | 2 | Semantic vs. Rest | MNI | -56 22 2  54 24 18  -46 30 -6  50 24 -6  -42 -18 -12  -52 -44 22  -36 -28 8  -54 -54 32 |
| 30276 | 1999 | Dapretto M | Neuron | 8 | 3 | Syntactic vs. Semantic | MNI | -44 22 10 |
| 30276 | 1999 | Dapretto M | Neuron | 8 | 4 | Semantic vs. Syntactic | MNI | -48 20 -4 |
| 7040096 | 2006 | Ethofer T | NeuroImage | 24 | 2 | Emotional Word Content > Affective Prosody | MNI | -21 36 51  -48 9 -39  -48 30 -6 |
| 16050116 | 2009 | Haupt S | Human Brain Mapping | 29 | 2 | Incongruent Semantic > Congruent Semantic | MNI | 2 -56 26 |
| 30145 | 2001 | Just M A | NeuroImage | 18 | 1 | Sentence Comprehension vs. Fixation | Talairach | -52 -19 6  51 -21 5  -29 -52 44  29 -54 42  -49 -16 11  49 -14 12  3 -72 8  -26 -70 3  32 -67 4  -34 25 35  32 33 35  0 13 35  -37 18 18  40 21 13  -40 -1 45  38 2 41  -1 16 48  -35 -8 49  37 -5 45  1 -2 56  1 -55 -12 |
| 30145 | 2001 | Just M A | NeuroImage | 18 | 3 | Sum of Single Tasks | Talairach | -52 -19 6  51 -21 5  -29 -52 44  29 -54 42  -49 -16 11  49 -14 12  3 -72 8  -26 -70 3  32 -67 4  -34 25 35  32 33 35  0 13 35  -37 18 18  40 21 13  -40 -1 45  38 2 41  -1 16 48  -35 -8 49  37 -5 45  1 -2 56  1 -55 -12 |
| 30145 | 2001 | Just M A | NeuroImage | 18 | 4 | Dual Task | Talairach | -52 -19 6  51 -21 5  -29 -52 44  29 -54 42  -49 -16 11  49 -14 12  3 -72 8  -26 -70 3  32 -67 4  -34 25 35  32 33 35  0 13 35  -37 18 18  40 21 13  -40 -1 45  38 2 41  -1 16 48  -35 -8 49  37 -5 45  1 -2 56  1 -55 -12 |
| 30149 | 2000 | Kuperberg G R | Journal of Cognitive Neuroscience | 9 | 1 | Normal Sentences > Random Word Strings | Talairach | -40 -42 -7  -32 -44 9  46 -25 4  -43 -53 20  35 11 -2  -20 -78 -7  -12 -53 20  20 17 -13  -3 -67 -7  -12 -69 -13  -6 17 -2 |
| 30149 | 2000 | Kuperberg G R | Journal of Cognitive Neuroscience | 9 | 2 | Random Word Strings > Normal Sentences | Talairach | -12 3 -18  46 6 -13  -17 -28 -13  6 -36 -2  -38 -53 -18  40 -50 -13  52 -44 -7  -46 -25 -2  -40 -47 15  -52 -22 15  -40 -6 -7  3 -69 -2  0 -50 -18  6 17 -2  -6 -14 -7 |
| 30149 | 2000 | Kuperberg G R | Journal of Cognitive Neuroscience | 9 | 3 | Pragmatically Violated Sentences > Normal Sentences | Talairach | -49 -31 9  0 -69 9  9 -83 15  -12 -39 9  -29 -36 -13  -32 14 -2  43 6 4  -6 -67 -7 |
| 30149 | 2000 | Kuperberg G R | Journal of Cognitive Neuroscience | 9 | 4 | Normal Sentences > Pragmatically Violated Sentences | Talairach | 29 3 -18  -38 8 -13  26 -22 -7  -35 -31 -2  -40 -33 -7  -35 -53 -2  -40 -56 9  0 -78 -2  -23 25 -2  14 3 -7 |
| 30149 | 2000 | Kuperberg G R | Journal of Cognitive Neuroscience | 9 | 5 | Semantically Violated Sentences > Normal Sentences | Talairach | 43 -11 -7  49 -17 4 |
| 30149 | 2000 | Kuperberg G R | Journal of Cognitive Neuroscience | 9 | 6 | Normal Sentences > Semantically Violated Sentences | Talairach | -49 -44 -7  -32 -72 -7  -9 -72 4  20 -64 -2  12 -61 -2  12 -81 4  12 -67 9  -43 -8 4  26 -69 -13  12 -64 -7  -29 -69 -13  26 -6 4  0 -56 -2 |
| 30149 | 2000 | Kuperberg G R | Journal of Cognitive Neuroscience | 9 | 7 | Syntactically Violated Sentences > Normal Sentences | Talairach | -43 -31 -7 |
| 30149 | 2000 | Kuperberg G R | Journal of Cognitive Neuroscience | 9 | 8 | Normal Sentences > Syntactically Violated Sentences | Talairach | -26 -14 -13  12 -31 -2  -17 -33 -2  -38 -42 -2  -35 -69 9  -49 -53 9  49 -14 -2  -46 -47 15  46 22 -2  20 -53 4  20 53 -2  -3 -78 -2  -9 -53 4  -14 -50 9  14 -50 15  9 -56 -7  -12 -22 9 |
| 30149 | 2000 | Kuperberg G R | Journal of Cognitive Neuroscience | 9 | 9 | (Normal vs. Pragmatic) > (Normal vs. Semantic and Syntax) | Talairach | -43 -31 9  -12 -39 9  3 -72 9  9 -81 15 |
| 30149 | 2000 | Kuperberg G R | Journal of Cognitive Neuroscience | 9 | 10 | (Normal vs. Semantic) > (Normal vs. Syntax) | Talairach | 43 -19 4  49 -8 -7  -43 -8 4 |
| 30091 | 2000 | Le Clec'H G | NeuroImage | 5 | 1 | Body Parts > Numbers (Block) | MNI | -39 -63 45  -51 9 42  -6 18 51  -48 45 21 |
| 30091 | 2000 | Le Clec'H G | NeuroImage | 6 | 3 | Body Parts > Numbers (ER) | MNI | -36 -69 45  -45 15 42  6 27 48 |
| 13030030 | 2006 | Lee S S | NeuroImage | 12 | 1 | Literal > Rest | Talairach | -62 -20 2  -60 -30 6  -56 -22 10  -46 12 10  -50 -42 6  -42 24 -10  -46 18 -6  22 -50 -16  -4 -46 -12  -8 -74 -8  52 -20 10  46 -24 10  50 -20 -4  54 -6 -2  54 2 -4  -52 2 42  -54 8 28  -54 -6 38  -6 4 52  38 12 -2  44 22 2 |
| 13030030 | 2006 | Lee S S | NeuroImage | 12 | 2 | Nonliteral > Rest | Talairach | -60 -28 4  -58 -34 8  -48 -22 10  -42 -26 -8  54 -10 2  40 -28 10  44 -22 -2  56 -32 12  -42 -4 40  -52 18 28  -54 18 32  6 -90 16  -8 -80 18  -6 80 6  6 88 4  -42 30 50  -54 -36 36  -48 18 -4  -36 22 -10  -4 12 48 |
| 13030030 | 2006 | Lee S S | NeuroImage | 12 | 3 | Nonliteral > Literal | Talairach | -50 20 28  -46 -60 48  -60 -8 -6 |
| 5040054 | 2004 | Maguire E A | NeuroImage | 12 | 1 | Facts vs. Baseline | MNI | -30 -15 -18  -51 -9 -21  -51 -66 18  -54 -51 30  12 -87 -27  -42 27 -9  0 -12 12  -45 21 30  -39 30 6 |
| 5040054 | 2004 | Maguire E A | NeuroImage | 12 | 2 | Control vs. Baseline | MNI | -42 36 -12  -48 24 24 |
| 5040054 | 2004 | Maguire E A | NeuroImage | 12 | 3 | Facts vs. Control | MNI | -54 3 36  -54 24 6  -48 30 -12  3 36 30  -6 -84 -24  0 -15 6  -51 -54 -21 |
| 30156 | 2001 | Michael E B | Human Brain Mapping | 9 | 2 | Auditory Object Relative - Baseline | Talairach | -52 -29 5  53 -24 6  -46 -20 11  49 -16 13  -43 15 24  41 16 20  -44 -49 -12  43 -44 -9  3 -66 12  -34 -61 37  43 -46 30  -12 -61 49  26 -64 49  -34 30 31  37 28 32  -41 -3 47  45 -2 50  -4 -12 64 |
| 7040106 | 2003 | Mitchell R L C | Neuropsychologia | 13 | 8 | Semantics vs. Prosody | Talairach | -21 -72 3  48 6 0  -48 -51 51  0 -84 6  -3 -90 30  -15 -36 66  -30 -24 -9  -39 0 57  -3 -6 3  -54 -27 -18  -24 -69 54  -48 18 33  -60 -18 39 |
| 7040106 | 2003 | Mitchell R L C | Neuropsychologia | 13 | 10 | Attention to semantics induced activity positively covarying with performance accuracy | Talairach | -54 -54 18  60 -42 21  -12 39 12  -54 -39 24  54 -9 21  24 30 36  -36 36 18 |
| 9020045 | 2004 | Mitchell R L C | British Journal of Psychiatry | 12 | 4 | Attention to Semantics, Normals > Schizophrenia Patients | MNI | -36 33 21  48 30 12 |
| 5080189 | 2001 | Poldrack R A | Journal of Cognitive Neuroscience | 8 | 2 | Compression-Related Decrease | MNI | 40 -14 16  6 -68 20  -26 0 24  -46 -32 12  32 -50 -8  -46 -70 20  46 -12 -4 |
| 5080189 | 2001 | Poldrack R A | Journal of Cognitive Neuroscience | 8 | 3 | Compression-Related Increase | MNI | -28 54 16  34 26 -4  4 32 20  18 4 8  66 -40 8 |
| 5080189 | 2001 | Poldrack R A | Journal of Cognitive Neuroscience | 8 | 4 | Convex Compression Response | MNI | -64 -24 0  -52 22 12  -34 14 20  36 26 8  -38 34 8 |
| 5080189 | 2001 | Poldrack R A | Journal of Cognitive Neuroscience | 8 | 5 | Convex Compression Response and Rhyme > Lettercase | MNI | -54 18 8  -38 12 20  14 8 4 |
| 5080189 | 2001 | Poldrack R A | Journal of Cognitive Neuroscience | 8 | 6 | Increasing Compression Response and Rhyme > Lettercase | MNI | -40 32 4  14 8 4 |
| 11010024 | 2006 | Saur D | Brain | 14 | 1 | Intelligible Speech > Reversed Speech, Healthy Controls | MNI | -60 -45 3  -42 12 24  -45 24 -6  33 24 -6  54 27 21  -57 -9 -15  -42 -45 -18  -36 -66 24  9 18 48 |
| 11010024 | 2006 | Saur D | Brain | 14 | 8 | Healthy Controls > Aphasic Patients, Acute Phase | MNI | -42 -45 -18  -42 12 24  33 24 -3  -63 -51 3  -57 -12 -12  -30 21 -3 |
| 11010024 | 2006 | Saur D | Brain | 14 | 9 | Aphasic Patients (Subacute Phase) > Healthy Controls | MNI | 36 30 -12  -9 33 42  -45 36 -9 |
| 7030084 | 2004 | Schirmer A | NeuroImage | 12 | 1 | Word Presentation > Rest, Females | Talairach | -23 -7 35  -14 -16 18  -52 -37 18  47 -22 12  31 -52 -18 |
| 7030084 | 2004 | Schirmer A | NeuroImage | 12 | 2 | Word Presentation > Rest, Males | Talairach | -19 -16 15  -44 -28 9  -26 -64 -18  49 -10 3  14 -55 -18 |
| 30334 | 2003 | Thierry G | Human Brain Mapping | 10 | 3 | Release Semantic | MNI | -42 -32 14  -48 -42 21  -48 -12 7  -40 12 7  -10 -22 7  -12 -10 14  -10 -32 0  -16 -72 0  -6 -82 21  -42 11 7  40 -36 14  50 -12 7  44 -38 21  8 -22 7  12 -28 0  18 -62 0  6 -80 0  39 18 7 |
| 30334 | 2003 | Thierry G | Human Brain Mapping | 10 | 4 | Hold Semantic | MNI | -48 -22 14  -40 -34 14  -48 -44 21  -50 -12 7  -50 0 0  -38 14 7  -30 18 7  -10 -22 7  -8 -30 0  -12 -8 14  -6 -62 0  -39 16 7  -48 -22 14  42 -36 14  48 8 0  46 -20 7  10 -20 7  12 -10 14  16 -62 0  2 -76 7  43 10 7 |
| 5040024 | 1999 | Thompson-Schill S L | Neuropsychologia | 5 | 1 | Living: Visual - Baseline | Talairach | -41 -53 -11  41 -34 4  -34 19 -4  -41 -4 34  -11 0 41 |
| 5040024 | 1999 | Thompson-Schill S L | Neuropsychologia | 5 | 2 | Living: Non-Visual - Baseline | Talairach | -45 -45 -8  -34 15 -4 |
| 5040024 | 1999 | Thompson-Schill S L | Neuropsychologia | 5 | 3 | Non-Living: Visual - Baseline | Talairach | -41 -53 -11  49 -30 4  -30 19 -4  -38 -4 34 |
| 5040024 | 1999 | Thompson-Schill S L | Neuropsychologia | 5 | 4 | Non-Living: Non-Visual - Baseline | Talairach | -49 -45 -4  53 -30 4  -30 19 0  -38 -4 38  11 4 34 |
| 11010031 | 2004 | Zahn R | Cognitive Brain Research | 14 | 1 | [Sem-Lex] & [Sem-Pho] Conjunction Analysis, Activations, Healthy Controls | MNI | -40 20 28  -36 -64 42  -52 -16 -14  -56 -8 28  44 -8 42  8 -76 0 |
| 11010031 | 2004 | Zahn R | Cognitive Brain Research | 14 | 2 | [Lex-Pho] & [Sem - Pho] Conjunction Analysis, Activations, Healthy Controls | MNI | -48 16 -7  -60 -16 -14  60 -8 -14  28 -32 28  4 44 42  12 4 14 |
