# Supplementary material for: Parcellation‐based anatomic model of the semantic network
Source: Brain Behav. 2021 Feb 18;11(4):e02065. doi: 10.1002/brb3.2065 (PMC8035438; doi:10.1002/brb3.2065)
Supplement: Supplementary file 2 — Table S2 [file BRB3-11-e02065-s002.docx]

Table S2. Studies Related to the Visual Words Task Paradigm

| **Brain Map ID** | **Year** | **First Author** | **Journal** | **Subjects** | **Experiment Number** | **Experiment Name** | **Coordinate**  **Space** | **Coordinates** |
| --- | --- | --- | --- | --- | --- | --- | --- | --- |
| 30424 | 2002 | Cansino S | Cerebral Cortex | 17 | 1 | Correct vs. Incorrect Source Memory During Encoding | MNI | -20 42 48  -4 30 62  -38 4 18  52 -6 54  -48 -4 54  -38 -10 60  0 -36 62  -44 -42 32  -58 -54 4  -42 -52 2  50 -70 0  28 -74 -10  38 -46 -12  -38 -54 -24  -14 16 -12  -10 -56 -18 |
| 16030067 | 2011 | Dennis N A | Neurobiology of Aging | 12 | 5 | Age x Task | Talairach | -23 -30 -11  23 -30 -11  19 1 21  26 -3 11 |
| 16010016 | 2014 | Fairhall S L | Cerebral Cortex | 17 | 7 | Word-cued Semantic Access, Person | MNI | 3 -49 28  3 53 -14 |
| 16010016 | 2014 | Fairhall S L | Cerebral Cortex | 17 | 8 | Word-cued Semantic Access, Place | MNI | -39 -79 34  -27 -32 -20  -15 -55 16  30 -31 -20  18 -52 16 |
| 7050138 | 2003 | Hariri A R | Biological Psychiatry | 11 | 2 | Label Pictures vs. Match Forms | MNI | 45 18 -5  -45 18 -5  -45 23 17  0 24 40  23 48 -10  -23 48 -10 |
| 7050138 | 2003 | Hariri A R | Biological Psychiatry | 11 | 4 | Label Pictures > Match Pictures | MNI | -38 22 -5  38 18 -5  -49 22 22  0 24 40 |
| 16050117 | 2009 | Heim S | Human Brain Mapping | 16 | 1 | Congruent > Null | MNI | -32 -14 -6  26 -4 6  -14 -24 2  10 -14 0  -54 -2 16  -60 -8 2  -36 -20 4  -50 6 2  4 -38 -6  -30 -60 -10  -40 -40 -14  -42 -82 -8  -34 -92 -2  -20 -68 -12  20 -62 0  22 -48 -12  -16 -66 12  6 -64 16  -6 -76 24  20 -64 20  10 14 58  -6 32 32  6 28 32  -18 -76 48  -28 -86 36  -20 -88 26  28 -52 64  28 -40 64  10 -68 -14  22 -82 36  18 -80 26  14 -30 56  12 -26 50  -30 26 6  -12 2 66  10 4 70  -42 4 36  -62 10 14  46 14 14  48 -28 58 |
| 16050117 | 2009 | Heim S | Human Brain Mapping | 16 | 2 | Incongruent > Null | MNI | -12 -22 2  10 -12 0  -50 4 4  -62 -2 4  -44 4 38  -54 -2 16  -36 10 8  -66 -28 12  -30 -4 -12  2 -40 -2  -34 -60 -10  -26 -92 -12  -38 -86 22  -22 -82 24  22 -80 38  -14 -68 12  6 -66 16  -6 -76 22  6 -82 32  -18 -76 48  -24 -62 -2  10 -86 -4  24 -72 -6  26 -42 -22  10 14 58  -12 4 68  2 24 38  8 -70 -16  -2 -60 2  32 -46 60  22 2 4  28 -88 30  32 -86 30  -40 -44 -14  26 -92 2  24 -94 4  -38 -56 -46  -40 14 30  26 -70 50 |
| 16050117 | 2009 | Heim S | Human Brain Mapping | 16 | 3 | Incongruent > Congruent | MNI | -50 -8 46  14 -40 -48  -32 36 38 |
| 16050117 | 2009 | Heim S | Human Brain Mapping | 16 | 4 | Congruent > Incongruent | MNI | 6 56 -4 |
| 16050117 | 2009 | Heim S | Human Brain Mapping | 16 | 5 | Onset Latency Difference for 1st Temporal Derivative of the HRF: Congruent > Incongruent | MNI | -50 -8 46  -46 -32 26  -34 -22 22 |
| 17060113 | 2010 | Hu W | Brain | 10 | 1 | Semantic word matching, Chinese > English; Control readers | MNI | -46 6 30  -56 -38 6 |
| 17060113 | 2010 | Hu W | Brain | 11 | 2 | Semantic word matching, Dyslexic > Control; Chinese speakers | MNI | -54 -38 4 |
| 17060113 | 2010 | Hu W | Brain | 10 | 3 | Semantic word matching, Dyslexic > Control; English speakers | MNI | -48 6 32 |
| 17060113 | 2010 | Hu W | Brain | 10 | 4 | Semantic word matching, English controls | MNI | -48 6 32  -54 -38 4 |
| 17060113 | 2010 | Hu W | Brain | 14 | 6 | Semantic word matching, Chinese controls | MNI | -48 6 32 |
| 9090122 | 2008 | Ino T | Open Neuroimaging Journal | 18 | 1 | Word > Color | MNI | -42 -86 -8  42 -80 -6  -28 -88 -10  32 -92 -6  -48 -68 -12  46 -80 -10  -24 -94 -2  30 -96 2  -44 -50 -22  50 -60 -16  -54 -58 -12 |
| 9090122 | 2008 | Ino T | Open Neuroimaging Journal | 18 | 2 | Color > Word | MNI | 50 10 26 |
| 30145 | 2001 | Just M A | NeuroImage | 18 | 3 | Sum of Single Tasks | Talairach | -52 -19 6  51 -21 5  -29 -52 44  29 -54 42  -49 -16 11  49 -14 12  3 -72 8  -26 -70 3  32 -67 4  -34 25 35  32 33 35  0 13 35  -37 18 18  40 21 13  -40 -1 45  38 2 41  -1 16 48  -35 -8 49  37 -5 45  1 -2 56  1 -55 -12 |
| 30145 | 2001 | Just M A | NeuroImage | 18 | 4 | Dual Task | Talairach | -52 -19 6  51 -21 5  -29 -52 44  29 -54 42  -49 -16 11  49 -14 12  3 -72 8  -26 -70 3  32 -67 4  -34 25 35  32 33 35  0 13 35  -37 18 18  40 21 13  -40 -1 45  38 2 41  -1 16 48  -35 -8 49  37 -5 45  1 -2 56  1 -55 -12 |
| 7020055 | 2005 | Katzir T | NeuroImage | 12 | 3 | Object Identity > Plus-Minus | MNI | -42 -72 -12  21 -99 -3  -36 24 -9  27 -33 -3  42 36 -12  -3 39 45  57 33 18  -30 -27 -12  9 24 51 |
| 8040105 | 2006 | Kensinger E A | Journal of Neuroscience | 21 | 1 | Pictures Corresponding with Subsequent Item-and-Source Memory | MNI | -30 11 51  45 46 -12  -24 26 -11  41 15 -23  45 -9 -9  -30 -12 -12  39 -21 -12  45 -24 -19  6 -17 12 |
| 8040105 | 2006 | Kensinger E A | Journal of Neuroscience | 21 | 3 | All Items Corresponding with Subsequent Item-and-Source Memory | MNI | -9 10 61  24 -47 55  39 13 -28  -36 -15 -14  15 -35 -6  -18 -6 -5 |
| 8040105 | 2006 | Kensinger E A | Journal of Neuroscience | 21 | 4 | Pictures Corresponding with Subsequent Item-Not-Source Memory | MNI | -15 48 31  -48 37 -13  -35 -65 7  42 -65 -8  30 -73 -11  -21 -10 -23  28 -1 -23  -40 -81 8 |
| 8040105 | 2006 | Kensinger E A | Journal of Neuroscience | 21 | 6 | All Items Corresponding with Item-Not-Source Memory | MNI | -3 20 43  -42 31 29  33 35 1  -27 -63 28  45 -67 6  24 -79 -9  36 -76 -11  42 -64 -2  -27 -73 -1  -30 -82 -6  -36 -62 -17  -37 -10 -25  30 -4 -23  -42 -78 12  42 -78 7  15 -79 -6  18 -98 10  -6 -51 -25 |
| 11010011 | 2008 | Kinno R | Human Brain Mapping | 14 | 2 | Canonical/Subject-Initial Active Sentence > Fixation | MNI | -45 6 48  48 21 33  -51 18 27  -51 27 6  -3 18 51  -6 3 3  9 3 0  -54 -54 6  51 -54 6  -24 -78 33  45 -78 6 |
| 11010011 | 2008 | Kinno R | Human Brain Mapping | 14 | 3 | Noncanonical/Subject-Inital Passive Sentence > Fixation | MNI | -39 -3 45  45 18 36  -51 21 24  -54 27 3  3 18 51  9 3 3  -54 -54 6  51 -60 9  -39 -57 54  -30 -81 30  42 -78 6 |
| 11010011 | 2008 | Kinno R | Human Brain Mapping | 14 | 4 | Noncanonical/Object-Initial Scrambled Sentence > Fixation | MNI | -42 3 48  48 21 33  -51 21 18  -48 18 0  42 21 -6  -3 18 48  -9 0 3  -60 -48 9  54 -36 3  -36 -57 51  -30 -84 30  45 -75 6 |
| 11010011 | 2008 | Kinno R | Human Brain Mapping | 14 | 5 | Canonical/Subject-Initial Active Sentence - Sentence Control | MNI | -39 0 54  -57 -60 0  54 -60 0  -51 -75 6 |
| 11010011 | 2008 | Kinno R | Human Brain Mapping | 14 | 6 | Noncanonical/Object-Initial Scrambled Sentence - Canonical/Subject-Initial Active Sentence | MNI | -39 0 45  -52 21 21  -54 -54 3 |
| 11010011 | 2008 | Kinno R | Human Brain Mapping | 14 | 7 | (Scrambled Sentence - Active Sentence) vs. (Active Sentence - Sentence Control) | MNI | -51 21 18  -51 -51 3 |
| 11010011 | 2008 | Kinno R | Human Brain Mapping | 14 | 8 | Noncanonical/Subject-Initial Passive Sentence - Canonical/Subject-Initial Active Sentence | MNI | -48 24 21 |
| 13030021 | 2008 | Lauro L J | Cerebral Cortex | 22 | 1 | Conjunction Among All Conditions | MNI | -46 28 16  -52 16 28  -44 -12 60  -56 2 32  -32 20 0  -34 -30 52  -44 -42 48  -48 -24 16  -34 -52 0  -38 -52 -24  -34 -84 16  -46 -78 -4  -14 -22 8  -32 -68 -20  -4 56 4  -2 -2 56  -2 42 -4  38 -6 64  50 28 16  50 8 28  32 22 -4  10 -50 32  62 -4 0  54 -68 0  44 -52 -20  22 -76 36  44 -70 8  46 -76 -12  10 8 8  12 -16 4  34 -46 -28 |
| 13030021 | 2008 | Lauro L J | Cerebral Cortex | 22 | 2 | Idiomatic > Literal | MNI | -4 54 32  -10 48 48  -50 26 0  -50 -58 24  -52 4 -32  -52 6 -40  52 32 -8  46 12 -36  54 0 -24  52 -48 12 |
| 13030021 | 2008 | Lauro L J | Cerebral Cortex | 22 | 3 | Literal > Idiomatic | MNI | -58 -32 40  42 -46 60  58 -36 52  62 -28 36 |
| 30402 | 2003 | Neumann K | Journal of Fluency Disorders | 5 | 13 | Between-Group: PWS Before Therapy < PWNS | MNI | -52 34 -14  -40 18 18  -6 20 44 |
| 30402 | 2003 | Neumann K | Journal of Fluency Disorders | 5 | 14 | Between-Group: PWS After Therapy < PWNS | MNI | -52 34 -14  -40 18 18  -6 20 44 |
| 30402 | 2003 | Neumann K | Journal of Fluency Disorders | 5 | 15 | Between-Group: PWS at Follow-up < PWNS | MNI | -52 36 4  -52 34 -14  -40 18 18  -6 20 44 |
| 30402 | 2003 | Neumann K | Journal of Fluency Disorders | 5 | 16 | Between-Group: PWS Over All Assessment Times < PWNS | MNI | -52 34 -14  -36 18 18 |
| 30402 | 2003 | Neumann K | Journal of Fluency Disorders | 5 | 17 | Between-Group: PWS Over All Assessment Times > PWNS | MNI | -46 12 44  -52 14 36  -48 22 6  -48 -64 -20 |
| 30410 | 2003 | Preibisch C | NeuroImage | 16 | 4 | PDS > Controls, Fixed effects further masked inclusive by fixed effects difference Semantic Decision | MNI | 36 18 -16 |
| 30410 | 2003 | Preibisch C | NeuroImage | 16 | 7 | Controls > PDS, Fixed effects further masked inclusive by fixed effects difference Semantic Decision | MNI | -46 -2 32 |
| 7060156 | 2006 | Ries M L | NeuroImage | 14 | 3 | Cognitive Conjunction, Normals | MNI | 8 -60 24  4 -66 22 |
| 7070190 | 2007 | Soderlund H | NeuroImage | 12 | 5 | Object Pair Encoding, Alcohol = Placebo | Talairach | -5 8 55 |
| 7070190 | 2007 | Soderlund H | NeuroImage | 12 | 6 | Object Pair Encoding, Alcohol NOT= Placebo | Talairach | 43 33 15  36 -43 -21  -36 -46 -9  29 -46 -7  -29 -60 35  -54 -65 -4  -29 -71 -7  14 -92 -6  53 7 -2  -45 -7 29  -18 -24 41  8 -29 43  -2 -38 19  -15 5 48 |
| 30181 | 2002 | Vaidya C J | Neuropsychologia | 8 | 1 | Encoding - Pictures vs. Words | MNI | -17 -66 -8  25 -54 -7  37 -85 17 |
| 10080214 | 2009 | Ye Z | NeuroImage | 19 | 6 | Plausibility, Stroop Congruency and Flanker Congruency | MNI | 8 34 42  -44 20 28  -36 26 -14  -32 16 8  -44 -46 46  56 26 14 |
| 10080214 | 2009 | Ye Z | NeuroImage | 19 | 8 | Plausibility vs. Stroop Congruency and Flanker Congruency | MNI | -8 56 18  -40 -60 28 |
| 7110336 | 2006 | Yoon H W | Neuroscience Letters | 14 | 1 | Picture > Fixation | Talairach | -40 -52 -16  40 -66 -18  -46 18 34 |
| 7110336 | 2006 | Yoon H W | Neuroscience Letters | 14 | 3 | Chinese Character > Picture | Talairach | 20 -8 16  -8 -20 36  -42 -18 36  -6 12 58 |
| 7110336 | 2006 | Yoon H W | Neuroscience Letters | 14 | 4 | Picture > Chinese Character | Talairach | 38 -62 -14  -28 -64 -14 |
| 30239 | 2001 | Zubicaray G I | Human Brain Mapping | 8 | 1 | Semantically Related Distractor vs. Control | MNI | -56 -20 -14  62 4 -16  -46 -46 16  10 62 2  -4 42 -4  -30 60 0  18 28 58  -54 -34 40  8 -76 -14 |
