# Supplementary material for: Parcellation‐based anatomic model of the semantic network
Source: Brain Behav. 2021 Feb 18;11(4):e02065. doi: 10.1002/brb3.2065 (PMC8035438; doi:10.1002/brb3.2065)
Supplement: Supplementary file 3 — Table S3 [file BRB3-11-e02065-s001.docx]

Table S3. Studies Related to the Visual Image Stimuli Paradigm

| **Brain Map ID** | **Year** | **First Author** | **Journal** | **Subjects** | **Experiment Number** | **Experiment Name** | **Coordinate**  **Space** | **Coordinates** |
| --- | --- | --- | --- | --- | --- | --- | --- | --- |
| 8080205 | 2006 | Addis D R | NeuroImage | 12 | 1 | Successful Encoding > Baseline Triad | Talairach | -61 9 16  40 26 6  46 28 19  -12 14 44  -8 21 38  4 20 41  -16 -17 5  10 -13 3  -16 -31 -2  -44 -37 44  34 -46 45  -42 -53 -18  -46 -66 -7  -36 -61 -20  18 -76 -35 |
| 8080205 | 2006 | Addis D R | NeuroImage | 12 | 2 | Zero Link > One Link > Two Link | Talairach | -57 20 16  42 30 11  -50 4 40  30 53 8  18 55 8  -44 4 35  -6 -30 27  36 -69 22  -12 -76 -15 |
| 8080205 | 2006 | Addis D R | NeuroImage | 12 | 3 | Two Link > One Link > Zero Link | Talairach | -14 37 33  -36 -17 51  -12 -6 6  -22 -9 -16  -40 11 -7  -38 -72 28  -14 -49 28  -4 -31 44  -46 -36 50  46 -35 46  -55 -41 32  55 -59 31  16 -80 -4  -2 -43 -5  10 -49 -11 |
| 8080205 | 2006 | Addis D R | NeuroImage | 12 | 4 | One Link + Two Link > Zero Link | Talairach | 42 19 34  4 -7 17  59 -6 -10  32 -53 30  -12 -88 -2 |
| 7040118 | 2007 | Aparicio M | NeuroImage | 12 | 1 | Visual Lexical Decision > Visual Feature Decision, Hearing Subjects | MNI | -52 20 18  44 26 6  -36 -20 60  -2 -2 68  -50 -22 -16  -62 -42 -2  -24 -94 -20  20 -86 30 |
| 7040118 | 2007 | Aparicio M | NeuroImage | 12 | 3 | Visual Lexical Decision, Hearing > Deaf Subjects | MNI | 62 -6 20  -4 -14 12  -50 14 -10  68 -16 30  56 -14 30 |
| 7040118 | 2007 | Aparicio M | NeuroImage | 12 | 4 | Visual Lexical Decision, Deaf > Hearing Subjects | MNI | 30 28 56  10 -40 16 |
| 8040092 | 2006 | Assaf M | Biological Psychiatry | 16 | 1 | Recall > No Recall, Normals | Talairach | -3 31 34  -3 38 1  -39 44 -2  56 26 4  -42 5 44  48 33 23  -53 -54 36  42 -51 33  9 -59 39  -62 -47 -5  -29 10 -26  56 -10 -22  -6 -12 1  9 6 0  -3 -23 -1 |
| 8040092 | 2006 | Assaf M | Biological Psychiatry | 16 | 3 | Recall > No Recall, Schizophrenic Patients > Normals | Talairach | -15 -6 53  12 5 44  3 15 -6  -32 -20 62  48 35 -4  30 22 32  -33 -81 7  50 58 1  -45 10 -28  27 7 -28  -30 -9 -12  27 -41 -3 |
| 8040092 | 2006 | Assaf M | Biological Psychiatry | 16 | 4 | Recall > No Recall, Normals > Schizophrenic Patients | Talairach | -38 -59 36  42 -56 33 |
| 8040099 | 2006 | Assaf M | Psychiatry Research | 18 | 1 | Correct Recall | Talairach | -3 16 46  -6 21 42  -36 -21 51  -42 6 24  -45 21 3  33 21 -6  -45 -33 48  -30 -53 44  -56 -44 -5  -24 -93 7  30 -85 -1  -9 -6 3  9 -12 9  -12 9 0  15 9 8 |
| 8040099 | 2006 | Assaf M | Psychiatry Research | 18 | 2 | Correct No Recall | Talairach | -3 17 49  -36 -18 48  -42 3 21  -44 17 2  42 20 -1  -42 -36 38  36 -47 41  -24 -59 47  33 -53 50  -24 -96 10  36 -90 5  -15 -20 9  12 -11 12  -12 6 5 |
| 8040099 | 2006 | Assaf M | Psychiatry Research | 18 | 3 | Correct Associated | Talairach | 6 16 43  -6 24 32  -41 -12 50  -38 10 27  -42 18 7  33 26 1  -33 -36 38  36 -44 46  -27 -50 41  30 -50 41  -51 -47 -7  68 -34 13  -27 -90 -3  42 -87 2  -15 -14 3  6 -17 4  12 12 -1  0 -18 -7 |
| 8040099 | 2006 | Assaf M | Psychiatry Research | 18 | 4 | Correct Non Associated | Talairach | 3 17 52  -3 20 46  -33 -12 59  -50 7 33  33 52 -5  -42 21 7  33 23 -6  -33 -39 41  33 -50 44  -30 -50 41  30 -50 47  -50 -53 -12  -36 -78 9  42 -87 7  -15 -11 3  6 -15 1  15 9 -3 |
| 8040099 | 2006 | Assaf M | Psychiatry Research | 18 | 5 | Correct Recall > No Recall | Talairach | -3 30 42  -3 41 3  -42 8 41  -42 32 -7  36 23 -9  -53 -48 33  -62 50 -5  -6 -6 3  15 9 8 |
| 8040099 | 2006 | Assaf M | Psychiatry Research | 18 | 6 | Correct Associated > Non Associated | Talairach | -12 41 6  -9 -39 35  -39 -50 44  -62 -52 -5 |
| 8040099 | 2006 | Assaf M | Psychiatry Research | 18 | 7 | Correct Non Associated > Associated | Talairach | 18 -10 42  18 13 35  -12 15 42 |
| 13070048 | 2009 | Aziz-Zadeh L | Human Brain Mapping | 12 | 1 | Aha Solutions > Search Solutions | MNI | -54 20 0  40 44 0  -6 26 44  38 12 -14  -48 -52 24  58 -52 36  42 6 -40  -6 -20 -26 |
| 13030019 | 2006 | Bedny M | Brain and Language | 13 | 1 | Words (Nouns + Verbs) > Non-words | MNI | -57 -48 0  -48 33 3  3 21 54  27 -66 9  -15 -90 0  39 15 0 |
| 13030019 | 2006 | Bedny M | Brain and Language | 13 | 2 | Non-words > Words (Nouns + Verbs) | MNI | -60 -30 36  -39 33 39  9 -54 42  -60 -24 -24  -60 -30 39  33 -12 9  15 30 57  45 -66 48  -45 -66 -9  -24 -3 -24  -54 -6 6 |
| 13030019 | 2006 | Bedny M | Brain and Language | 13 | 3 | Verbs > Nouns | MNI | -9 -36 21 |
| 13030019 | 2006 | Bedny M | Brain and Language | 13 | 4 | Positive Correlation between Imageability Ratings and Words (Nouns + Verbs) Activations | MNI | -27 -78 39  -21 18 54  45 -69 24 |
| 13030019 | 2006 | Bedny M | Brain and Language | 13 | 5 | Negative Correlation between Imageability Ratings and Words (Nouns + Verbs) Activations | MNI | -30 -99 -3  15 -90 -3 |
| 13030019 | 2006 | Bedny M | Brain and Language | 13 | 6 | Interaction between Imageability and Grammatical Class | MNI | -54 15 -6  -60 -51 0 |
| 5070126 | 2003 | Binder J R | Journal of Cognitive Neuroscience | 24 | 1 | Word > Nonword | Talairach | -25 15 55  -16 30 48  -7 49 27  -35 9 47  -31 21 34  0 46 13  -1 47 2  -9 40 -5  1 28 -12  -38 38 -5  -50 -64 26  -38 -74 40  -41 -76 27  -51 -48 43  -50 -65 14  -38 -62 52  -59 -47 -5  -59 -49 -15  -1 -38 35  -9 -60 12  -5 -58 41  -4 -54 27  1 -25 39  -7 -68 56  7 -93 4  -23 -10 -14 |
| 5070126 | 2003 | Binder J R | Journal of Cognitive Neuroscience | 24 | 2 | Nonword > Word | Talairach | -43 -1 24  -55 1 18  -51 -8 43  44 5 33  44 20 29  -1 3 51  0 13 45  27 49 19  -40 17 2 |
| 5070126 | 2003 | Binder J R | Journal of Cognitive Neuroscience | 24 | 3 | Lo > Hi | Talairach | -23 27 44  -15 40 30  -14 54 15  -14 30 49  -16 18 45  -3 57 11  -3 38 31  -22 27 34  13 52 17  -1 42 15  0 44 0  -7 29 -9  1 29 9  -5 1 4  -7 15 -3  7 5 5  -4 -7 11  45 -70 28  -42 -65 37  2 -37 35  4 -45 16  -1 -51 25  -7 -55 17  3 -67 38  -4 -55 62  -35 -46 -21 |
| 5070126 | 2003 | Binder J R | Journal of Cognitive Neuroscience | 24 | 4 | LoWord > HiWord | Talairach | -21 39 32  -26 26 45  -37 17 38  -30 9 46  -27 12 56  36 14 45  1 16 42  -43 -66 41  -50 -63 30  -48 -51 30  -58 -21 -11  -53 -13 -20  1 -40 31  1 -52 31 |
| 6060077 | 2005 | Bonner-Jackson A | Biological Psychiatry | 26 | 1 | Deep vs. Shallow Encoding, Normals | Talairach | -47 22 11  -13 13 62  -39 5 54  17 -31 -40  -62 -55 13  5 2 56  -48 33 -14  -53 -5 27  -45 18 43  -38 38 31 |
| 6060077 | 2005 | Bonner-Jackson A | Biological Psychiatry | 26 | 3 | Word > Face, Normals | Talairach | -39 26 3 |
| 6060077 | 2005 | Bonner-Jackson A | Biological Psychiatry | 26 | 9 | Deep Encoding vs. Fixation, Normals | Talairach | -2 1 55  -22 -91 -9  23 -86 -6  -49 10 26  -44 -34 52  -34 -75 -17  31 -60 -22  -42 25 11  -36 -54 -18  -25 6 8  -29 -63 47  -41 -9 56  44 30 32  32 17 6  -4 -26 0  32 -60 49  12 -72 -35  -6 -75 -25  -1 -47 42  4 -25 47  -45 -18 -1  -11 -63 22  10 -45 18  37 -7 -8  0 19 23  2 -81 32  -3 41 18  33 -61 19  -30 -47 19  -16 -44 60  48 -36 25  4 34 -3  51 -16 10  12 -54 64  21 -33 62  41 -17 40  -55 -34 19  -41 -74 25  -53 -54 31  16 46 29  -25 -46 -8  19 -45 -7  -39 -20 33  -31 -4 -14  20 20 45  -26 34 38 |
| 8050117 | 2007 | Bonner-Jackson A | Biological Psychiatry | 38 | 1 | Deep vs. Shallow Encoding, SNCs | Talairach | -52 27 -3  -22 -102 9  -2 12 60  26 -99 9  -44 42 9 |
| 8050117 | 2007 | Bonner-Jackson A | Biological Psychiatry | 21 | 2 | Deep vs. Shallow Encoding, SIBs | Talairach | -4 24 45  -46 12 27  40 24 0  32 -69 -36  -40 39 0  44 42 0 |
| 30079 | 2002 | Booth J R | Human Brain Mapping | 13 | 1 | Visual Meaning - Control | MNI | -3 36 36  -45 21 24  15 -72 15  21 -57 6  -15 15 3  -60 -51 0  21 -87 -3  -27 -90 -6  -27 -3 -6  42 21 -9  12 -54 -9  36 -69 -24  -36 -57 -27  15 -78 -30  0 -60 -33 |
| 30079 | 2002 | Booth J R | Human Brain Mapping | 13 | 5 | Visual Meaning - Rhyming | MNI | -42 9 36  51 33 18  -51 -45 -3 |
| 30079 | 2002 | Booth J R | Human Brain Mapping | 13 | 9 | Meaning - Rhyming | MNI | -6 18 42  -54 21 18  48 30 18  12 -81 12  -27 -63 0  18 -84 -3  -48 -33 -6  -48 21 -9 |
| 7110316 | 2007 | Carreiras M | Journal of Cognitive Neuroscience | 36 | 1 | (Lexical Decision + Read) > False Fonts | MNI | -28 -92 -4  14 -78 16  -40 -66 -12  -42 -42 -20  -52 -40 12  -62 -34 4  -62 -14 6  -52 10 -14  -44 24 -6  -54 12 0  -36 24 0  34 18 4  -44 -34 46  -24 -62 36  -20 -68 44  -26 -52 48  -60 -18 28  -48 6 32  -54 0 42  -46 -6 54  -26 -8 56  -4 4 58  8 6 56  8 18 44  -2 -78 -26  6 -74 -20  25 -68 -24  -28 -62 -24  -24 -6 6  -26 2 -4  -20 6 4  26 10 4 |
| 7110316 | 2007 | Carreiras M | Journal of Cognitive Neuroscience | 36 | 2 | Lexical Decision > False Fonts | MNI | -28 -92 -4  14 -78 16  -40 -66 -12  -42 -42 -20  -52 -40 12  -62 -34 4  -62 -14 6  -52 10 -14  -44 24 -6  -54 12 0  -36 24 0  34 18 4  -44 -34 46  -24 -62 36  -20 -68 44  -26 -52 48  -60 -18 28  -48 6 32  -54 0 42  -46 -6 54  -26 -8 56  -4 4 58  8 6 56  8 18 44  -2 -78 -26  6 -74 -20  25 -68 -24  -28 -62 -24  -24 -6 6  -26 2 -4  -20 6 4  26 10 4 |
| 7110316 | 2007 | Carreiras M | Journal of Cognitive Neuroscience | 36 | 4 | [Lexical Decision-False Fonts] > [Read-False Fonts], Lexical Decision > Read | MNI | -50 -22 54  34 18 4 |
| 7110316 | 2007 | Carreiras M | Journal of Cognitive Neuroscience | 36 | 5 | [Lexical Decision-False Fonts] > [Read-False Fonts], Lexical Decision > False Fonts | MNI | -50 -22 54  34 18 4 |
| 7110316 | 2007 | Carreiras M | Journal of Cognitive Neuroscience | 36 | 6 | [Lexical Decision-False Fonts] > [Read-False Fonts], Read > False Fonts | MNI | 34 18 4 |
| 7110316 | 2007 | Carreiras M | Journal of Cognitive Neuroscience | 36 | 7 | [Read-False Fonts] > [Lexical Decision-False Fonts], Lexical Decision > Read | MNI | -62 -14 6  -62 -34 4  56 -28 0 |
| 7110316 | 2007 | Carreiras M | Journal of Cognitive Neuroscience | 36 | 8 | [Read-False Fonts] > [Lexical Decision-False Fonts], Lexical Decision > False Fonts | MNI | -62 -14 6  -62 -34 4 |
| 7110316 | 2007 | Carreiras M | Journal of Cognitive Neuroscience | 36 | 10 | Lexical Decision > Read, (Words + Pseudowords + False Fonts) | MNI | 60 -20 46  -52 -28 48  -64 -26 28  -54 -22 18  -34 -32 52  24 -50 -26  2 -48 -32  -4 -12 54  30 -98 -10  6 8 -12  14 12 -16  -6 6 -12 |
| 7110316 | 2007 | Carreiras M | Journal of Cognitive Neuroscience | 36 | 11 | Lexical Decision > Read, Words | MNI | 60 -20 46  -52 -28 48  -64 -26 28  -54 -22 18  -34 -32 52  24 -50 -26  2 -48 -32  -4 -12 54  30 -98 -10  6 8 -12  14 12 -16  -6 6 -12 |
| 7110316 | 2007 | Carreiras M | Journal of Cognitive Neuroscience | 36 | 16 | Pseudowords > Words, (Lexical Decision + Read + False Fonts) | MNI | 0 10 56  6 18 44  -2 -4 60  50 22 22  -50 -4 50  -56 0 34  -46 8 28 |
| 7110316 | 2007 | Carreiras M | Journal of Cognitive Neuroscience | 36 | 17 | Pseudowords > Words, Lexical Decision | MNI | 0 10 56  6 18 44  -2 -4 60  50 22 22  -50 -4 50  -56 0 34  -46 8 28 |
| 7110316 | 2007 | Carreiras M | Journal of Cognitive Neuroscience | 36 | 22 | Words > Pseudowords, (Lexical Decision + Read + False Fonts) | MNI | 54 -70 20  -46 -74 38  -52 -68 16  -6 -46 32  -2 58 4 |
| 7110316 | 2007 | Carreiras M | Journal of Cognitive Neuroscience | 36 | 23 | Words > Pseudowords, Lexical Decision | MNI | 54 -70 20  -46 -74 38  -52 -68 16  -6 -46 32  -2 58 4 |
| 16030053 | 2006 | Carreiras M | Human Brain Mapping | 16 | 1 | Lexical Decision, Low Frequency Words (LFW) > High Frequency Words (HFW) | MNI | -46 18 22  -4 22 42  -2 10 58 |
| 16030053 | 2006 | Carreiras M | Human Brain Mapping | 16 | 2 | Interaction with Task, LFW > HFW | MNI | -46 18 22  -4 22 42  -2 10 58 |
| 16030053 | 2006 | Carreiras M | Human Brain Mapping | 16 | 3 | Lexical Decision-LFW, High Frequency Syllables (HFS) > Low Frequency Syllables (LFS) | MNI | -42 -12 -32 |
| 16030054 | 2009 | Carreiras M | Human Brain Mapping | 20 | 1 | Low Frequency - High Frequency | Talairach | -3 17 45  9 -55 40  -37 28 4  42 17 4 |
| 16030054 | 2009 | Carreiras M | Human Brain Mapping | 20 | 2 | Incongruent - Congruent | Talairach | 3 -47 52  -7 -22 10  10 -22 10  3 46 10 |
| 10030085 | 2008 | Chan S W Y | Neuropsychologia | 10 | 1 | Negative Words vs. Control Words | MNI | 14 -84 6  -4 56 24  -44 28 -10  -2 18 42 |
| 10030085 | 2008 | Chan S W Y | Neuropsychologia | 10 | 2 | Positive Words vs. Control Words | MNI | 14 -82 4  -56 28 -2 |
| 10030085 | 2008 | Chan S W Y | Neuropsychologia | 10 | 3 | Positive Words vs. Negative Words | MNI | 66 -18 30 |
| 10030085 | 2008 | Chan S W Y | Neuropsychologia | 10 | 4 | Negative Words vs. Positive Words | MNI | -24 14 66 |
| 10030085 | 2008 | Chan S W Y | Neuropsychologia | 10 | 5 | Negative Words > Positive Words, High N Scores vs. Low N Scores | MNI | 20 -64 64 |
| 10030085 | 2008 | Chan S W Y | Neuropsychologia | 10 | 6 | Positive Correlations with N, Negative Words | MNI | -8 -8 52  -20 -28 66 |
| 10030085 | 2008 | Chan S W Y | Neuropsychologia | 10 | 7 | Positive Correlations with N, Positive Words | MNI | -46 26 -6 |
| 10030085 | 2008 | Chan S W Y | Neuropsychologia | 10 | 10 | Correlations with Categorization and Recognition of Negative Words | MNI | -10 26 46 |
| 12020002 | 2003 | Chee M W | Human Brain Mapping | 12 | 1 | Low and High Frequency, Block Design, Size | Talairach | -40 13 27  35 16 26  32 22 8  -2 1 47  -55 -44 -3  -43 -56 -12  -31 -65 38 |
| 12020002 | 2003 | Chee M W | Human Brain Mapping | 8 | 2 | Low and High Frequency, Block Design, Fixation | Talairach | -46 7 30  37 6 30  -43 -31 40  -4 3 51  -26 -57 46  24 -65 40  -40 -70 -9 |
| 12020002 | 2003 | Chee M W | Human Brain Mapping | 12 | 3 | Low and High Frequency, Event-Related Design | Talairach | -37 12 27  38 14 30  28 23 9  35 1 30  -6 7 45  -45 -50 -9  -31 -64 44  28 -67 39  -28 -69 -9  -16 -62 4 |
| 12020002 | 2003 | Chee M W | Human Brain Mapping | 12 | 4 | Low > High Frequency, Block Design, Size | Talairach | -40 15 28  -46 27 17  32 22 8  -4 14 44 |
| 12020002 | 2003 | Chee M W | Human Brain Mapping | 8 | 5 | Low > High Frequency, Block Design, Fixation | Talairach | -42 15 28  -46 29 21 |
| 30426 | 2001 | Chee M W L | NeuroImage | 10 | 1 | English Words - Control, SGP | Talairach | -5 29 44  -36 7 30  -38 28 4  -24 -62 35  -44 -54 -13 |
| 30426 | 2001 | Chee M W L | NeuroImage | 9 | 2 | English Words - Control, PRC | Talairach | -4 12 48  -40 6 32  -41 19 6  29 24 6  -24 -77 31  -49 -58 -9 |
| 30426 | 2001 | Chee M W L | NeuroImage | 10 | 5 | Mandarin Characters > English Words, SGP | Talairach | -3 25 41  -37 6 30  -29 24 4  -26 -64 45  -41 -59 -17 |
| 30426 | 2001 | Chee M W L | NeuroImage | 9 | 6 | English Words > Mandarin Characters, PRC | Talairach | -3 8 48  -39 4 30  -31 23 4  30 24 6 |
| 16030057 | 1999 | Chee M W L | Human Brain Mapping | 8 | 4 | Visual Abstract/Concrete > Fixation | Talairach | 40 51 3  25 54 3  -31 51 0  40 27 31  -28 18 18  53 30 21  50 15 3  -46 9 31  -46 12 18  -40 33 12  -43 3 28  -40 -6 56  34 -27 53  6 3 65  6 3 53  -3 21 62  -2 -9 65  -43 -45 43  -37 -42 34  40 -60 -21  21 -81 -15  -34 -60 -12  -25 -84 -15  9 -69 -28  0 -51 -31  -18 -42 -25  -18 -78 -18  -21 -54 -18  18 6 21 |
| 16030057 | 1999 | Chee M W L | Human Brain Mapping | 8 | 5 | Abstract/Concrete > Syllable | Talairach | 31 54 3  53 42 6  -43 42 0  -40 36 15  34 18 -3  -28 18 15  -37 15 -3  50 36 -6  -46 30 0  -46 18 34  -46 9 21  -40 -6 46  -6 9 65  -6 24 43  -28 6 50  -15 3 34  -56 -36 0  -59 -6 -15  -37 -54 -9  28 -57 -37  15 -30 -28  15 -69 -34  -34 -45 -21  -25 -60 -31  -18 -30 -31 |
| 16030057 | 1999 | Chee M W L | Human Brain Mapping | 8 | 6 | Abstarct/Concrete > Case | Talairach | 31 51 3  -34 48 3  -31 21 15  -43 33 9  -40 15 31  -37 27 0  -25 33 0  40 -6 56  -3 9 62  12 -63 -37 |
| 16030058 | 2008 | Chen E | Brain and Language | 14 | 1 | Predicate Metaphors > Literal Motion Sentences | Talairach | -53 18 7  -50 7 -26  -53 -9 -15  -62 -38 7  -42 -57 33  53 -1 -25 |
| 16030058 | 2008 | Chen E | Brain and Language | 14 | 2 | Literal Motion Sentences < Non-Motive Sentences | Talairach | -53 -1 -15  -24 -53 -2  -9 -84 10  53 8 -16  24 -47 -3  9 -75 9 |
| 16030058 | 2008 | Chen E | Brain and Language | 14 | 3 | Predicate Metaphors > Non-Motive Sentences | Talairach | -56 -49 11 |
| 16030058 | 2008 | Chen E | Brain and Language | 14 | 4 | Positive Correlation between Figurativeness Ratings and Activations Across All Sentences | Talairach | -56 29 4  -50 7 -26  -53 -9 -15  -53 -40 8  -42 -56 36  50 2 -18 |
| 16030062 | 2006 | Chou T L | Human Brain Mapping | 35 | 1 | Related Words > Baseline | Talairach | -51 5 41  33 23 -1  -6 23 43  -39 -36 -16  3 -69 12  -21 -29 -1 |
| 16030062 | 2006 | Chou T L | Human Brain Mapping | 35 | 2 | Unrelated Words > Baseline | Talairach | -33 29 -6  33 26 -4  -6 17 43  -51 -41 6  15 -69 12  -23 -44 55  42 -6 58  -54 -19 22  53 -9 50  -24 -26 1  9 -18 -9 |
| 16030062 | 2006 | Chou T L | Human Brain Mapping | 35 | 3 | Related Words > Unrelated Words | Talairach | -30 -65 42  -30 17 54  -6 -35 29 |
| 16030062 | 2006 | Chou T L | Human Brain Mapping | 35 | 4 | Correlation between Activation and Higher Semantic Association in Related Word Pairs | Talairach | -18 -68 45  48 -52 11  45 -59 39  -33 56 6  33 31 32  -40 -50 44 |
| 16030062 | 2006 | Chou T L | Human Brain Mapping | 35 | 5 | Correlation between Activation and Lower Semantic Association in Related Word Pairs | Talairach | -53 -44 5  -53 16 5 |
| 16030062 | 2006 | Chou T L | Human Brain Mapping | 35 | 6 | Correlation between Activation and Increasing Age in Related Words | Talairach | -45 -33 40  -30 -10 42  -53 -50 0 |
| 16030062 | 2006 | Chou T L | Human Brain Mapping | 35 | 7 | Correlation between Activation and Increasing Age in Unrelated Words | Talairach | -39 -21 48  24 6 60 |
| 16030062 | 2006 | Chou T L | Human Brain Mapping | 35 | 8 | Correlation between Deactivation and Age in Related Words | Talairach | 1 52 0  56 -20 1  6 -46 13 |
| 16030062 | 2006 | Chou T L | Human Brain Mapping | 35 | 9 | Correlation between Deactivation and Age in Unrelated Words | Talairach | 53 -23 4  -56 -12 -2  -62 -26 13  12 -9 45 |
| 16030062 | 2006 | Chou T L | Human Brain Mapping | 35 | 10 | Correlation between Activation and Increasing Accuracy in Related Words, p<0.001 | Talairach | -9 -18 42 |
| 16030062 | 2006 | Chou T L | Human Brain Mapping | 35 | 11 | Correlation between Activation and Increasing Accuracy in Related Words, p<0.005 | Talairach | -60 -45 41 |
| 16030062 | 2006 | Chou T L | Human Brain Mapping | 35 | 12 | Correlation between Activation and Decreasing Accuracy in Related Words | Talairach | 65 -24 -5 |
| 16030062 | 2006 | Chou T L | Human Brain Mapping | 35 | 13 | Correlation between Activation and Longer Response Time in Unrelated Words | Talairach | 10 36 19 |
| 17040076 | 2014 | Christodoulou J A | PLoS ONE | 12 | 1 | Fast>Medium>Slow, Typical Readers | Talairach | -2 8 70  30 26 0  -50 -46 8  54 -46 14  -34 -76 -4  36 -88 18  48 -78 4  -38 -46 -38  -8 -78 -38  -8 -16 4  12 12 8  4 -34 -46 |
| 17040076 | 2014 | Christodoulou J A | PLoS ONE | 12 | 2 | Medium > Slow, Typical Readers | Talairach | -2 8 60  48 26 26  34 32 -2  38 -4 38  50 -40 6  -40 -24 48  36 -46 50  30 -68 36  18 -80 -2 |
| 14010002 | 2012 | Contreras J M | Social Cognitive and Affective Neuroscience | 19 | 1 | Categorical Knowledge (Non-Social > Social) | MNI | -46 -36 44  -58 -60 -4  6 12 22  -48 6 18  14 -18 68  42 -10 24  -44 38 12  -24 10 56 |
| 14010002 | 2012 | Contreras J M | Social Cognitive and Affective Neuroscience | 19 | 2 | Feature Verification (Object > Person) | MNI | -48 -52 -12  -40 32 14 |
| 14010002 | 2012 | Contreras J M | Social Cognitive and Affective Neuroscience | 19 | 3 | Categorical Knowledge (Social > Non-Social) | MNI | -4 -58 28  -8 56 34  -4 48 -8  -50 -10 -22  60 -2 -22  -56 -60 24  56 -56 18  -12 -96 -4  -26 -74 -16  -10 38 50 |
| 14010002 | 2012 | Contreras J M | Social Cognitive and Affective Neuroscience | 19 | 4 | Feature Verification (Person > Object) | MNI | -2 58 22  -56 -4 -24  38 22 -22  -56 -64 26  54 -12 -32  -40 20 -16  -4 0 6  44 30 -8  62 18 16 |
| 30345 | 2002 | Cooke A | Human Brain Mapping | 7 | 1 | Subject-Relative Short - Baseline | MNI | -68 -44 8  4 -84 -16 |
| 30345 | 2002 | Cooke A | Human Brain Mapping | 7 | 2 | Subject-Relative Long - Baseline | MNI | -68 -44 12  60 -24 -4  0 -88 -8 |
| 30345 | 2002 | Cooke A | Human Brain Mapping | 7 | 3 | Object-Relative Short - Baseline | MNI | -64 -56 8 |
| 30345 | 2002 | Cooke A | Human Brain Mapping | 7 | 4 | Object-Relative Long - Baseline | MNI | -64 -56 8  52 -68 16  60 -20 -8  -52 28 -8  -32 -20 -20  0 -92 -8 |
| 30345 | 2002 | Cooke A | Human Brain Mapping | 7 | 5 | Object-Relative Long - Subject-Relative Short | MNI | -52 28 -8  52 -68 16  0 -92 -8 |
| 30345 | 2002 | Cooke A | Human Brain Mapping | 7 | 6 | Object-Relative Long - Object-Relative Short | MNI | -56 12 -4  48 -64 16 |
| 30345 | 2002 | Cooke A | Human Brain Mapping | 7 | 7 | Subject-Relative Long - Object-Relative Short | MNI | -56 -24 0 |
| 30345 | 2002 | Cooke A | Human Brain Mapping | 7 | 8 | Subject-Relative Long - Subject-Relative Short | MNI | 56 -24 0 |
| 30345 | 2002 | Cooke A | Human Brain Mapping | 7 | 9 | Object-Relative Short - Subject-Relative Short | MNI | -48 -68 -8  -4 -92 -8  28 -68 -20 |
| 30345 | 2002 | Cooke A | Human Brain Mapping | 7 | 10 | Object-Relative Long - Subject-Relative Long | MNI | -40 -76 -4  -32 -20 -20  36 -40 -12  16 -92 -12 |
| 30345 | 2002 | Cooke A | Human Brain Mapping | 7 | 11 | Object-Relative Short - Subject-Relative Long | MNI | -4 -56 -12  -8 -36 16 |
| 30431 | 2001 | Curtis V A | Journal of Affective Disorders | 5 | 7 | Bipolars vs. Controls, Semantic Decision | Talairach | 26 -86 -2  40 -67 -13  20 -72 -7  -23 -75 -7 |
| 30432 | 1999 | Curtis V A | Schizophrenia Research | 5 | 1 | Semantic Decision - Control, Normals | Talairach | -14 -83 -2  14 -86 9  -3 -64 26  20 -78 -2  3 -56 48  -55 -25 20  43 -69 -7  -20 -69 42  0 -39 37  -35 -53 -13  -46 8 37  35 -58 -13  -49 17 26  46 -64 26  43 -50 42  49 14 16  52 6 48  9 -24 42 |
| 30432 | 1999 | Curtis V A | Schizophrenia Research | 5 | 3 | Normals vs. Patients | Talairach | -17 -81 -7  20 -78 4  -32 -83 4  14 -86 9  26 -83 15  43 -58 -13  -38 -53 -13  3 3 59  -46 -64 -2  -29 -72 -13  -43 3 48 |
| 30432 | 1999 | Curtis V A | Schizophrenia Research | 5 | 4 | Group x Task Interactions | Talairach | -17 -81 -7  20 -78 4  0 -47 48  6 -58 20  14 -86 9  -46 6 42  -46 14 4  -29 -72 -13  32 -58 -13  -38 -53 -13  3 6 53  26 -81 20 |
| 7110317 | 2007 | Curtis V A | Bipolar Disorders | 12 | 1 | Overall Task Effect, Bipolar Patients > Normals | Talairach | 18 -78 -24  -40 -4 42 |
| 7110317 | 2007 | Curtis V A | Bipolar Disorders | 12 | 2 | Overall Task Effect, Normals > Bipolar Patients | Talairach | 4 11 42 |
| 7110317 | 2007 | Curtis V A | Bipolar Disorders | 12 | 4 | Semantic Tasks > Repetition + Word Form Analysis | Talairach | -43 7 31 |
| 11080061 | 2008 | Davis M H | Brain and Language | 12 | 1 | All Words vs. Letter Strings | MNI | -63 -42 -3  -45 -42 -12  -6 27 -42  -48 30 -15  -33 27 -12  -48 24 18  -18 -9 -15  9 27 42  33 -12 60 |
| 11080061 | 2008 | Davis M H | Brain and Language | 12 | 2 | (Complex Noun vs. Simple Noun) vs. (Complex Verb vs. Simple Verb) | MNI | -30 -6 18  -27 48 -12 |
| 11080061 | 2008 | Davis M H | Brain and Language | 12 | 3 | Verbs (Simple/Complex) vs. Nouns (Simple/Complex) | MNI | -54 -48 -6  -54 -36 21  -21 -36 3  -3 9 -6 |
| 30239 | 2001 | de Zubicaray G I | Human Brain Mapping | 8 | 1 | Semantically Related Distractor vs. Control | MNI | -56 -20 -14  62 4 -16  -46 -46 16  10 62 2  -4 42 -4  -30 60 0  18 28 58  -54 -34 40  8 -76 -14 |
| 30080 | 2001 | Dehaene S | Nature Neuroscience | 37 | 3 | Same vs. Different Word, Same Case | MNI | 32 -80 -16  24 -88 4 |
| 30080 | 2001 | Dehaene S | Nature Neuroscience | 37 | 4 | Same vs. Different Word, Independent of Case | MNI | -44 -52 -20  -44 4 28  44 4 28 |
| 16030067 | 2011 | Dennis N A | Neurobiology of Aging | 12 | 1 | High Confidence Hits > Misses, Explicit Learning, Younger Healthy Controls | Talairach | -49 9 24  -26 -34 -14  -23 -42 44  -56 -52 -10  -23 -72 39  26 -92 5  -19 -85 -5 |
| 16030067 | 2011 | Dennis N A | Neurobiology of Aging | 12 | 2 | High Confidence Hits > Misses, Explicit Learning, Older Healthy Controls | Talairach | -41 30 9  -26 -34 -17  -23 29 -14  -49 13 34  -56 -44 6 |
| 16030067 | 2011 | Dennis N A | Neurobiology of Aging | 12 | 5 | Age x Task | Talairach | -23 -30 -11  23 -30 -11  19 1 21  26 -3 11 |
| 30347 | 2003 | Devlin J T | Journal of Cognitive Neuroscience | 12 | 1 | Semantic + Phonological - Rest | MNI | -44 8 28  -50 16 22  -46 30 16  -42 24 0  -42 24 2  -36 26 -6  -42 48 -14  -46 20 -6  -6 -2 62  -28 -72 50  -52 -48 -2  -44 -54 -20  -34 -42 -36  -36 -44 -28  -34 -6 -40  24 -98 -4  18 -92 -10  34 -90 -14  -24 -94 -6  -36 -92 -10  -16 -92 -12  26 -50 -38  34 -46 -40  42 -46 -38  12 -78 -44  10 -62 -14 |
| 30347 | 2003 | Devlin J T | Journal of Cognitive Neuroscience | 12 | 2 | Semantic > Phonological | MNI | -10 52 -8  -14 44 -8  -20 30 48  -42 -66 28  -4 -56 28 |
| 13030029 | 2011 | Diaz M T | Journal of Cognitive Neuroscience | 16 | 1 | All Valid Sentences > Nonword Sentences | MNI | -54 28 2  34 26 -6  -38 20 14  28 20 -4  -10 58 24  -60 -32 2  50 -18 -8 |
| 13030029 | 2011 | Diaz M T | Journal of Cognitive Neuroscience | 16 | 2 | Metaphor > Literal | MNI | -34 16 -18  -8 56 32  -58 -18 -14  -58 -42 4  -8 -12 42  30 -98 12 |
| 13030029 | 2011 | Diaz M T | Journal of Cognitive Neuroscience | 16 | 3 | Congruent > Incongruent | MNI | -46 34 -8  52 26 10  36 2 46  -6 34 46  6 52 -24  44 22 -22  -60 -40 -8  -40 -52 28  30 -92 8  0 -28 -10 |
| 13030029 | 2011 | Diaz M T | Journal of Cognitive Neuroscience | 16 | 4 | Incongruent > Congruent | MNI | -60 -4 -4  -46 -22 24  -38 -26 68 |
| 7090260 | 2003 | Ding G S | Neuroreport | 6 | 6 | English Semantic vs. Asterisks | Talairach | -44 49 -21  57 32 44  -60 30 -15  52 -18 31  -31 46 52 |
| 7090260 | 2003 | Ding G S | Neuroreport | 6 | 7 | Chinese Semantic vs. Asterisks - English Semantic vs. Asterisks | Talairach | -59 -6 -9  -35 -15 57 |
| 7090260 | 2003 | Ding G S | Neuroreport | 6 | 8 | English Semantic vs. Asterisks - Chinese Semantic vs. Asterisks | Talairach | 55 -25 31  56 -3 30  59 -17 31 |
| 7100287 | 2007 | Eddington K M | Journal of Cognitive Neuroscience | 16 | 1 | Self + Other Relevance vs. Syllable Counting | Talairach | -3 30 15  -48 23 -12  3 54 31  -48 -60 32  42 -54 32 |
| 7100287 | 2007 | Eddington K M | Journal of Cognitive Neuroscience | 16 | 2 | Self vs. Other Relevance | Talairach | -18 -52 11  0 42 31  30 0 63  -48 -26 1  45 -24 0  -33 6 -4 |
| 7100287 | 2007 | Eddington K M | Journal of Cognitive Neuroscience | 16 | 3 | Promotion Goal Priming | Talairach | -36 40 -17  45 34 -17  27 -6 -11 |
| 7100287 | 2007 | Eddington K M | Journal of Cognitive Neuroscience | 16 | 4 | Prevention Goal Priming | Talairach | 9 29 -1 |
| 8100242 | 2004 | Elliott R | Biological Psychiatry | 8 | 1 | All Go/NoGo vs. Rest; Normals > Manic Patients | MNI | -45 6 36 |
| 8100242 | 2004 | Elliott R | Biological Psychiatry | 8 | 2 | All Go/NoGo vs. Rest; Manic Patients > Normals | MNI | -60 -12 6  63 -6 3 |
| 8100242 | 2004 | Elliott R | Biological Psychiatry | 8 | 3 | Semantic vs. Orthographic Conditions; Normals > Manic Patients | MNI | -54 -12 -21  45 -18 -30  33 -93 -6  -54 33 -6  51 30 -12 |
| 8100242 | 2004 | Elliott R | Biological Psychiatry | 8 | 4 | Semantic vs. Orthographic Targets; Manic Patients > Normals | MNI | 12 -66 54  51 48 6  24 60 3 |
| 16010016 | 2014 | Fairhall S L | Cerebral Cortex | 17 | 7 | Word-cued Semantic Access, Person | MNI | 3 -49 28  3 53 -14 |
| 16010016 | 2014 | Fairhall S L | Cerebral Cortex | 17 | 8 | Word-cued Semantic Access, Place | MNI | -39 -79 34  -27 -32 -20  -15 -55 16  30 -31 -20  18 -52 16 |
| 9080081 | 2001 | Ferstl E C | Cognitive Brain Research | 12 | 1 | Language Conditions > Non-Word Control | Talairach | -10 15 52  -8 44 42  -50 -12 -7  -59 -49 13  -46 20 19  -46 28 5  -8 -54 23  44 27 11  43 -10 -8  18 -79 -20 |
| 9080081 | 2001 | Ferstl E C | Cognitive Brain Research | 12 | 2 | (Coherent/Incohesive + Coherent/Cohesive) - (Incoherent/Incohesive + Incoherent/Cohesive) | Talairach | -5 -34 39  -4 58 13 |
| 9080081 | 2001 | Ferstl E C | Cognitive Brain Research | 12 | 3 | (Coherent/Cohesive + Incoherent/Cohesive) - (Coherent/Incohesive + Incoherent/Incohesive) | Talairach | -25 0 34  24 1 37 |
| 9080081 | 2001 | Ferstl E C | Cognitive Brain Research | 12 | 4 | (Coherent/Incohesive + Incoherent/Cohesive) - (Coherent/Cohesive + Incoherent/Incohesive) | Talairach | -40 22 15 |
| 9080081 | 2001 | Ferstl E C | Cognitive Brain Research | 12 | 5 | Incoherent/Cohesive - Incoherent/Incohesive | Talairach | -44 9 19  -31 17 17 |
| 9080081 | 2001 | Ferstl E C | Cognitive Brain Research | 12 | 6 | Coherent/Cohesive - Coherent/Incohesive | Talairach | -28 -60 48  -45 -40 43 |
| 5070135 | 2002 | Fiebach C J | Journal of Cognitive Neuroscience | 12 | 1 | Words vs. Pseudowords | Talairach | -28 -34 -12  33 -36 -8  -32 -45 -7  35 -48 -10  -29 -89 1  32 -82 0  -24 -90 13  31 -81 19  -18 -98 9  22 -95 2  -37 -68 9  38 -64 10  -52 -52 11  26 -61 34 |
| 5070135 | 2002 | Fiebach C J | Journal of Cognitive Neuroscience | 12 | 2 | Low vs. High Frequency Words | Talairach | -51 31 10  -42 1 17  -29 28 -4  33 24 -2  -10 -10 7  18 -5 14 |
| 5070135 | 2002 | Fiebach C J | Journal of Cognitive Neuroscience | 12 | 3 | Pseudowords vs. High Frequency Words | Talairach | -47 10 13  -37 16 0  7 31 10  -3 -3 12 |
| 7020052 | 2005 | Fiebach C J | Human Brain Mapping | 14 | 1 | Long vs. Short Object | Talairach | -44 21 11  45 21 10  -46 17 4  -54 7 28  -54 -27 -1  -52 -46 6  45 -18 -3  -18 -18 12 |
| 7020052 | 2005 | Fiebach C J | Human Brain Mapping | 14 | 2 | Long vs. Short Subject | Talairach | -20 -52 22  19 -59 20  -14 -65 43 |
| 5080182 | 2002 | Gold B T | Neuron | 24 | 1 | Semantic Decision on Words | Talairach | -45 35 -4  -47 17 24  -51 -55 2 |
| 9020038 | 2003 | Grossman M | Brain | 16 | 1 | Animals and Implements minus Pseudowords, Normals | Talairach | -60 -40 20  48 -24 24 |
| 9020038 | 2003 | Grossman M | Brain | 11 | 3 | Animals and Implements minus Pseudowords, Normals > AD Patients | Talairach | -60 -36 20  -44 12 24  -32 -72 16  40 -28 -8  20 4 8 |
| 9020038 | 2003 | Grossman M | Brain | 11 | 4 | Animals and Implements minus Pseudowords, AD Patients > Normals | Talairach | -36 -28 -8 |
| 9020038 | 2003 | Grossman M | Brain | 11 | 5 | Animals minus Pseudowords, All Subjects | Talairach | -16 -12 0  32 24 8 |
| 9020038 | 2003 | Grossman M | Brain | 11 | 6 | Implements minus Pseudowords, All Subjects | Talairach | 4 12 8  16 32 16 |
| 9020038 | 2003 | Grossman M | Brain | 11 | 7 | Animals > Implements, All Subjects | Talairach | -20 -4 -8 |
| 9020038 | 2003 | Grossman M | Brain | 11 | 8 | Implements > Animals, All Subjects | Talairach | 16 24 24  4 12 8 |
| 9020038 | 2003 | Grossman M | Brain | 11 | 9 | Animals, Normals > AD Patients | Talairach | -52 -32 24 |
| 9020038 | 2003 | Grossman M | Brain | 11 | 10 | Animals, AD Patients > Normals | Talairach | -28 -40 -4 |
| 9020038 | 2003 | Grossman M | Brain | 11 | 11 | Implements, Normals > AD Patients | Talairach | -56 -40 12 |
| 9020038 | 2003 | Grossman M | Brain | 11 | 12 | Implements, AD Patients > Normals | Talairach | -52 4 20  20 32 16  -20 -12 0 |
| 9020038 | 2003 | Grossman M | Brain | 16 | 13 | Animals > Implements, Normals | Talairach | -20 -4 -8 |
| 16040107 | 2002 | Grossman M | Human Brain Mapping | 16 | 1 | All Verbs - Baseline | Talairach | -64 -48 12  -4 40 -12  0 -92 4 |
| 16040107 | 2002 | Grossman M | Human Brain Mapping | 16 | 2 | [Motion Verbs - Baseline] - [Cognition Verbs - Baseline] | Talairach | -8 -64 0  12 36 -12  16 16 20 |
| 16040107 | 2002 | Grossman M | Human Brain Mapping | 16 | 3 | [Cognition Verbs - Baseline] - [Motion Verbs - Baseline] | Talairach | -56 -68 8 |
| 16040108 | 2007 | Gu X | Behavioural Brain Research | 10 | 1 | Rating Painful Words > Counting Neutral Words | Talairach | 34 10 1  46 23 25  -38 33 8  -42 -32 20  -57 -12 26  -28 -74 4  22 -7 6 |
| 16040108 | 2007 | Gu X | Behavioural Brain Research | 10 | 2 | Rating Painful Words > Counting Painful Words | Talairach | 38 -9 8  46 21 23  -46 -44 8  -16 -21 42  -50 -14 32  48 -16 23  -61 -15 17  -28 -73 9 |
| 16040108 | 2007 | Gu X | Behavioural Brain Research | 10 | 3 | Conj. Analysis: (Rating Painful vs. Counting Neutral) vs. (Rating Painful vs. Counting Painful) | Talairach | 34 10 2  46 23 23  -44 -44 10  -50 -14 32  48 -16 23  -28 -74 4 |
| 5070138 | 2001 | Haist F | Brain and Language | 15 | 1 | PH with Words, Increases | Talairach | -50 -38 -19  -48 43 3  -66 -34 33 |
| 5070138 | 2001 | Haist F | Brain and Language | 15 | 2 | PH with Words, Decreases | Talairach | -54 -56 -13  -59 19 23 |
| 5070138 | 2001 | Haist F | Brain and Language | 15 | 3 | PH with Pseudohomophones, Increases | Talairach | -50 -53 -18  -54 35 5  -65 -50 30 |
| 5070138 | 2001 | Haist F | Brain and Language | 15 | 4 | PH with Pseudohomophones, Decreases | Talairach | -56 -58 -13  -49 32 8 |
| 5070138 | 2001 | Haist F | Brain and Language | 15 | 5 | PW with Words, Decreases | Talairach | -50 -62 -15 |
| 5070138 | 2001 | Haist F | Brain and Language | 15 | 6 | PW with Pseudowords, Increases | Talairach | -50 -42 -9 |
| 5070138 | 2001 | Haist F | Brain and Language | 15 | 7 | PW with Pseudowords, Decreases | Talairach | -61 -44 -13  -59 18 22  -60 -30 29 |
| 7100276 | 2007 | Haller S | European Journal of Neuroscience | 16 | 1 | Simple - Fixation | Talairach | 25 -81 -7  25 -81 -7  -38 -78 -5  40 -28 60  -50 7 42  0 5 49  30 -52 47  33 22 10  -57 -15 28  52 -13 30  -24 -57 47  -30 28 11  -46 15 12  1 -30 26 |
| 7100276 | 2007 | Haller S | European Journal of Neuroscience | 16 | 2 | Medium - Simple | Talairach | -36 -63 -2.7  -42 96 30  45 1.4 45  2.8 -2.3 60  24 -85 1.6  -29 -48 45  28 5.9 13  37 -46 -12  47 20 -.71  45 -23 11 |
| 7100276 | 2007 | Haller S | European Journal of Neuroscience | 16 | 3 | Complex - Medium | Talairach | -3 -65 6.5  -40 3.8 43  -1.7 3.4 58  -52 -39 11  31 -6.2 57  42 15 36  -31 25 14  -55 -3.6 -1.9  32 27 14  30 -51 39 |
| 12100066 | 2010 | Han S | Neuropsychologia | 20 | 1 | Death-Related > Neutral-Valence Words | MNI | -4 48 36  -6 -58 38  -32 -66 40  40 -64 28  44 16 38  -48 24 20 |
| 12100066 | 2010 | Han S | Neuropsychologia | 20 | 2 | Negative-Valence > Neutral-Valence Words | MNI | -6 42 22  -8 60 18  -12 -74 14  -8 -58 46  30 24 46  24 48 30  42 -30 12 |
| 12100066 | 2010 | Han S | Neuropsychologia | 20 | 3 | Neutral-Valence > Death-Related Words | MNI | -40 -4 2  48 -8 10 |
| 12100066 | 2010 | Han S | Neuropsychologia | 20 | 4 | Negative-Valence > Death-Related Words | MNI | 0 14 28  -40 -14 12  42 -8 8 |
| 14050062 | 2012 | Hargreaves I S | Brain and Language | 15 | 1 | Animal SCT > Concrete SCT | Talairach |  |
| 14050062 | 2012 | Hargreaves I S | Brain and Language | 15 | 2 | Concrete SCT > Animal SCT | Talairach |  |
| 16050117 | 2009 | Heim S | Human Brain Mapping | 16 | 1 | Congruent > Null | MNI | -32 -14 -6  26 -4 6  -14 -24 2  10 -14 0  -54 -2 16  -60 -8 2  -36 -20 4  -50 6 2  4 -38 -6  -30 -60 -10  -40 -40 -14  -42 -82 -8  -34 -92 -2  -20 -68 -12  20 -62 0  22 -48 -12  -16 -66 12  6 -64 16  -6 -76 24  20 -64 20  10 14 58  -6 32 32  6 28 32  -18 -76 48  -28 -86 36  -20 -88 26  28 -52 64  28 -40 64  10 -68 -14  22 -82 36  18 -80 26  14 -30 56  12 -26 50  -30 26 6  -12 2 66  10 4 70  -42 4 36  -62 10 14  46 14 14  48 -28 58 |
| 16050117 | 2009 | Heim S | Human Brain Mapping | 16 | 2 | Incongruent > Null | MNI | -12 -22 2  10 -12 0  -50 4 4  -62 -2 4  -44 4 38  -54 -2 16  -36 10 8  -66 -28 12  -30 -4 -12  2 -40 -2  -34 -60 -10  -26 -92 -12  -38 -86 22  -22 -82 24  22 -80 38  -14 -68 12  6 -66 16  -6 -76 22  6 -82 32  -18 -76 48  -24 -62 -2  10 -86 -4  24 -72 -6  26 -42 -22  10 14 58  -12 4 68  2 24 38  8 -70 -16  -2 -60 2  32 -46 60  22 2 4  28 -88 30  32 -86 30  -40 -44 -14  26 -92 2  24 -94 4  -38 -56 -46  -40 14 30  26 -70 50 |
| 16050117 | 2009 | Heim S | Human Brain Mapping | 16 | 3 | Incongruent > Congruent | MNI | -50 -8 46  14 -40 -48  -32 36 38 |
| 16050117 | 2009 | Heim S | Human Brain Mapping | 16 | 4 | Congruent > Incongruent | MNI | 6 56 -4 |
| 16050117 | 2009 | Heim S | Human Brain Mapping | 16 | 5 | Onset Latency Difference for 1st Temporal Derivative of the HRF: Congruent > Incongruent | MNI | -50 -8 46  -46 -32 26  -34 -22 22 |
| 6080118 | 2003 | Hofer A | American Journal of Psychiatry | 10 | 1 | Comparison Subjects, Encoding vs. Rest | MNI | -40 6 22  -40 38 0  42 4 27  34 38 -4  24 48 0  -22 42 4  -48 -48 9 |
| 6080118 | 2003 | Hofer A | American Journal of Psychiatry | 10 | 3 | Comparisons - Schizophrenics, Encoding vs. Rest | MNI | 18 50 18  22 40 22  14 30 18  -46 -42 4 |
| 6080119 | 2003 | Hofer A | American Journal of Psychiatry | 10 | 1 | Comparisons > Schizophrenics, Encoding | MNI | 24 53 6  24 53 -3  -12 -31 35  20 -56 16  -10 -52 11  -16 -69 20 |
| 17060113 | 2010 | Hu W | Brain | 10 | 1 | Semantic word matching, Chinese > English; Control readers | MNI | -46 6 30  -56 -38 6 |
| 17060113 | 2010 | Hu W | Brain | 11 | 2 | Semantic word matching, Dyslexic > Control; Chinese speakers | MNI | -54 -38 4 |
| 17060113 | 2010 | Hu W | Brain | 10 | 3 | Semantic word matching, Dyslexic > Control; English speakers | MNI | -48 6 32 |
| 17060113 | 2010 | Hu W | Brain | 10 | 4 | Semantic word matching, English controls | MNI | -48 6 32  -54 -38 4 |
| 17060113 | 2010 | Hu W | Brain | 14 | 6 | Semantic word matching, Chinese controls | MNI | -48 6 32 |
| 7120385 | 2007 | Ilg R | NeuroImage | 15 | 1 | (Coherent Explicit + Coherent Implicit + Incoherent) > Pseudowords | MNI | 0 8 56  0 16 40  -8 26 38  -4 24 30  -52 10 24  54 18 -6  -46 10 32  -34 20 -4  40 20 -2  50 12 30  -28 -70 54  -52 -44 -6  -44 -62 -20  -14 -82 8  10 -70 12  16 -50 4  -2 -14 10  8 -2 6  6 -78 -16  -12 -84 -18  40 -68 -24 |
| 7120385 | 2007 | Ilg R | NeuroImage | 15 | 2 | Coherent Implicit > Incoherent | MNI | -6 4 64  4 2 66  -2 24 26  -52 16 -4  56 10 -8  -24 50 28  -2 -68 60  42 -66 -24 |
| 7120385 | 2007 | Ilg R | NeuroImage | 15 | 3 | Coherent Implicit > Coherent Explicit | MNI | 58 -40 24  -52 -56 40  46 -28 -10 |
| 13060042 | 2008 | Izuma K | Neuron | 19 | 2 | Interaction: (Self - Other) x (HSR - NoSR) | MNI | -14 52 8  -36 50 10  -6 40 18  -14 24 54  -8 24 32  -52 20 14  -22 20 -2  22 16 -4  -4 -22 30  -44 -32 -8  -62 -18 -10  16 -48 -38  -6 -58 -12 |
| 13060042 | 2008 | Izuma K | Neuron | 19 | 3 | Main Effect: Self > Other | MNI | 12 2 -2  -6 2 6  -46 -54 -12  -34 -94 -10  50 -56 -16  40 -84 -6 |
| 13060042 | 2008 | Izuma K | Neuron | 19 | 4 | Main Effect: Other > Self | MNI | 34 28 38  36 -4 -46  28 -24 -26  -32 -6 -42  -20 -14 -22  -36 -16 16  -50 -38 20  10 -72 -4  -6 -88 -12  4 -30 62  6 -56 50  10 -102 16 |
| 9110179 | 2002 | Kelley W M | Journal of Cognitive Neuroscience | 21 | 1 | All Trials > Fixation | MNI | -24 -94 -12  32 -92 -4  0 -70 -12  -4 -46 4  -2 -78 40  -32 -66 54  30 -64 50  -48 12 22  48 8 32  -42 16 -4  -32 26 -8  54 16 -6  42 0 58  0 14 42  -6 -20 8  -10 4 6  34 -62 -32  -28 -60 -32 |
| 9110179 | 2002 | Kelley W M | Journal of Cognitive Neuroscience | 21 | 2 | Fixation > All Trials | MNI | 10 52 2  -8 54 2  6 40 44  -4 4 -10  18 34 52  34 26 46  14 58 28  -14 50 26  -24 36 -10  12 -48 50  -56 -58 38  -34 -40 -12  -16 -84 18  -44 -76 30  24 -8 -18  56 -24 -14 |
| 9110179 | 2002 | Kelley W M | Journal of Cognitive Neuroscience | 21 | 3 | Self Relevance + Other Relevance > Case | MNI | -42 16 -4  0 14 42 |
| 9110179 | 2002 | Kelley W M | Journal of Cognitive Neuroscience | 21 | 4 | Self Relevance > Other Relevance | MNI | 10 52 2  12 -48 50 |
| 12070055 | 2008 | Kemmerer H | Brain and Language | 16 | 1 | Running Verbs > Wingdings | MNI | -28 -32 60  24 -32 62  -60 -36 0  -54 24 28  -54 32 16  -46 16 36  -44 30 -16  -6 44 46  -60 -42 22  -58 -52 18  -60 -4 -12  -50 -22 -10  -46 -14 -30  46 12 -18  58 -6 -12  60 2 -10  24 -48 72  14 -84 26  12 -70 20  10 -40 22  -34 -30 -6  -30 -42 2  -36 -20 24 |
| 12070055 | 2008 | Kemmerer H | Brain and Language | 16 | 2 | Speaking Verbs > Wingdings | MNI | -54 -38 2  -48 -30 -8  -50 18 20  -50 24 -12  -46 34 -2  -4 56 36  -2 48 -14  34 -40 2 |
| 12070055 | 2008 | Kemmerer H | Brain and Language | 16 | 3 | Hitting Verbs > Wingdings | MNI | -28 -30 62  14 -26 62  10 -34 68  -54 -38 2  -66 -36 2  -58 -34 -10  -46 40 4  -32 40 -14  -42 46 -14  -58 -4 -14  60 4 -8  24 -48 72  14 -58 32  0 28 -20 |
| 12070055 | 2008 | Kemmerer H | Brain and Language | 16 | 4 | Cutting Verbs > Wingdings | MNI | -46 28 24  -46 10 40  -54 -40 2  -60 -42 16  60 -58 -8  -44 -60 12  -46 -14 -30  -36 -68 42  -38 -60 38  -52 36 18  -58 16 -12  -28 38 -12  -50 38 -2  -54 26 -4  -4 48 -18  -36 40 18  70 -8 16  54 -72 26  24 -50 72  10 -58 30  16 -66 12  10 -70 20  44 -16 4 |
| 12070055 | 2008 | Kemmerer H | Brain and Language | 16 | 5 | Change of State Verbs > Wingdings | MNI | -56 -38 0  -44 -16 -28  -52 -26 -22  -46 18 22  -38 30 22  -4 46 50  -58 -6 -14  -58 -44 18  10 -68 18 |
| 8040105 | 2006 | Kensinger E A | Journal of Neuroscience | 21 | 2 | Words Corresponding with Subsequent Item-and-Source Memory | MNI | 22 -47 60  46 3 4  32 15 -21  -52 -35 -3  -30 -12 -15 |
| 8040105 | 2006 | Kensinger E A | Journal of Neuroscience | 21 | 3 | All Items Corresponding with Subsequent Item-and-Source Memory | MNI | -9 10 61  24 -47 55  39 13 -28  -36 -15 -14  15 -35 -6  -18 -6 -5 |
| 8040105 | 2006 | Kensinger E A | Journal of Neuroscience | 21 | 5 | Words Corresponding with Subsequent Item-Not-Source Memory | MNI | 33 44 -2  17 -4 39  2 18 17  -36 -62 -20  35 -76 -10  -37 -12 -26  30 3 -20  35 -75 -20 |
| 8040105 | 2006 | Kensinger E A | Journal of Neuroscience | 21 | 6 | All Items Corresponding with Item-Not-Source Memory | MNI | -3 20 43  -42 31 29  33 35 1  -27 -63 28  45 -67 6  24 -79 -9  36 -76 -11  42 -64 -2  -27 -73 -1  -30 -82 -6  -36 -62 -17  -37 -10 -25  30 -4 -23  -42 -78 12  42 -78 7  15 -79 -6  18 -98 10  -6 -51 -25 |
| 30035 | 2002 | Kiehl K A | NeuroImage | 28 | 1 | Congruent vs. Incongruent Terminal Word Endings | MNI | -48 32 4  -32 32 -16  -48 16 20  -48 -52 -16  -48 -60 -20  -48 -44 -24  24 -16 68  -40 -16 60  -32 -20 64  36 32 -16  36 12 -16  28 32 -24 |
| 11010011 | 2008 | Kinno R | Human Brain Mapping | 14 | 2 | Canonical/Subject-Initial Active Sentence > Fixation | MNI | -45 6 48  48 21 33  -51 18 27  -51 27 6  -3 18 51  -6 3 3  9 3 0  -54 -54 6  51 -54 6  -24 -78 33  45 -78 6 |
| 11010011 | 2008 | Kinno R | Human Brain Mapping | 14 | 3 | Noncanonical/Subject-Inital Passive Sentence > Fixation | MNI | -39 -3 45  45 18 36  -51 21 24  -54 27 3  3 18 51  9 3 3  -54 -54 6  51 -60 9  -39 -57 54  -30 -81 30  42 -78 6 |
| 11010011 | 2008 | Kinno R | Human Brain Mapping | 14 | 4 | Noncanonical/Object-Initial Scrambled Sentence > Fixation | MNI | -42 3 48  48 21 33  -51 21 18  -48 18 0  42 21 -6  -3 18 48  -9 0 3  -60 -48 9  54 -36 3  -36 -57 51  -30 -84 30  45 -75 6 |
| 11010011 | 2008 | Kinno R | Human Brain Mapping | 14 | 5 | Canonical/Subject-Initial Active Sentence - Sentence Control | MNI | -39 0 54  -57 -60 0  54 -60 0  -51 -75 6 |
| 11010011 | 2008 | Kinno R | Human Brain Mapping | 14 | 6 | Noncanonical/Object-Initial Scrambled Sentence - Canonical/Subject-Initial Active Sentence | MNI | -39 0 45  -52 21 21  -54 -54 3 |
| 11010011 | 2008 | Kinno R | Human Brain Mapping | 14 | 7 | (Scrambled Sentence - Active Sentence) vs. (Active Sentence - Sentence Control) | MNI | -51 21 18  -51 -51 3 |
| 11010011 | 2008 | Kinno R | Human Brain Mapping | 14 | 8 | Noncanonical/Subject-Initial Passive Sentence - Canonical/Subject-Initial Active Sentence | MNI | -48 24 21 |
| 30222 | 2002 | Kircher T T J | Neuropsychologia | 6 | 1 | Intentional Judgment of Personality Traits | Talairach | -6 -53 31  6 -53 37  -26 -47 48  -20 0 -7  -17 0 4  -49 -17 20  -17 -25 -2  -23 -67 9  -12 -22 31  -38 0 20 |
| 30222 | 2002 | Kircher T T J | Neuropsychologia | 6 | 2 | Incidental Judgment of Self Descriptors | Talairach | -46 3 31  40 -44 42  -43 -50 15  55 -31 -2  -12 -83 4  -30 -50 48 |
| 9090125 | 2008 | Knaus T A | Journal of the International Neuropsychological Society | 12 | 1 | Naming > Letter Strings, Typically Developing Adolescents | MNI | -50 25 14  -54 -11 -12  -58 -37 6  -32 -37 -16  -32 33 -12  -36 -13 -40  -44 -.5 50 |
| 9090125 | 2008 | Knaus T A | Journal of the International Neuropsychological Society | 12 | 3 | Naming > Letter Strings, ASD Adolescents > Typically Developing Adolescents | MNI | 62 31 20  72 -57 -6  -54 19 18  -62 -65 -8  -34 3 34  -58 -69 12  -2 5 60  -36 17 -.25  38 13 24  38 39 -14  42 -71 56 |
| 8050132 | 2003 | Kubicki M | NeuroImage | 9 | 1 | Deep Encoding, Normals | MNI | -52 26 16  42 22 -14  46 8 44  0 18 44  -22 -94 -4  36 -84 -6 |
| 8050132 | 2003 | Kubicki M | NeuroImage | 9 | 3 | Deep vs. Shallow Encoding, Normals | MNI | -48 46 -8  40 22 -18 |
| 8050132 | 2003 | Kubicki M | NeuroImage | 9 | 6 | Normals > Schizophrenics, Deep vs. Shallow Encoding | MNI | 46 48 -10 |
| 8050132 | 2003 | Kubicki M | NeuroImage | 9 | 7 | Schizophrenics > Normals, Deep vs. Shallow Encoding | MNI | -54 -22 8 |
| 8050132 | 2003 | Kubicki M | NeuroImage | 9 | 8 | Normals > Schizophrenics, Deep Encoding | MNI | 52 28 -2  -52 28 6 |
| 8050132 | 2003 | Kubicki M | NeuroImage | 9 | 9 | Schizophrenics > Normals, Deep Encoding | MNI | 0 10 36  -54 -6 18 |
| 9030055 | 2005 | Kuchinke L | NeuroImage | 20 | 1 | Words > Nonwords | MNI | -36 27 45  -15 57 9  -48 -69 30  -63 -21 -24  -63 -54 -9  -39 -69 42  -21 39 42  -39 -78 36  -12 57 39  -6 -30 33  -6 27 -18  54 -63 18  42 -69 33  12 -57 27 |
| 9030055 | 2005 | Kuchinke L | NeuroImage | 20 | 2 | Nonwords > Words | MNI | -42 27 0  -60 -9 -6  -9 3 60  -45 -51 -18  33 27 -6  51 24 21  24 -9 -21  27 15 15 |
| 13030021 | 2008 | Lauro L J | Cerebral Cortex | 22 | 1 | Conjunction Among All Conditions | MNI | -46 28 16  -52 16 28  -44 -12 60  -56 2 32  -32 20 0  -34 -30 52  -44 -42 48  -48 -24 16  -34 -52 0  -38 -52 -24  -34 -84 16  -46 -78 -4  -14 -22 8  -32 -68 -20  -4 56 4  -2 -2 56  -2 42 -4  38 -6 64  50 28 16  50 8 28  32 22 -4  10 -50 32  62 -4 0  54 -68 0  44 -52 -20  22 -76 36  44 -70 8  46 -76 -12  10 8 8  12 -16 4  34 -46 -28 |
| 13030021 | 2008 | Lauro L J | Cerebral Cortex | 22 | 2 | Idiomatic > Literal | MNI | -4 54 32  -10 48 48  -50 26 0  -50 -58 24  -52 4 -32  -52 6 -40  52 32 -8  46 12 -36  54 0 -24  52 -48 12 |
| 13030021 | 2008 | Lauro L J | Cerebral Cortex | 22 | 3 | Literal > Idiomatic | MNI | -58 -32 40  42 -46 60  58 -36 52  62 -28 36 |
| 30091 | 2000 | Le Clec'H G | NeuroImage | 5 | 1 | Body Parts > Numbers (Block) | MNI | -39 -63 45  -51 9 42  -6 18 51  -48 45 21 |
| 30091 | 2000 | Le Clec'H G | NeuroImage | 6 | 3 | Body Parts > Numbers (ER) | MNI | -36 -69 45  -45 15 42  6 27 48 |
| 12100070 | 2007 | Lieberman M D | Psychological Science | 30 | 1 | Gender Labeling > Affect Labeling | MNI | -24 0 -24  4 48 -16  6 8 -4  6 26 -8  2 18 32  44 -14 2  -40 -12 8  -8 -64 26  66 -42 14  -56 -22 12  40 6 -18  -40 8 -14  6 -26 -2  10 -54 -12 |
| 11110102 | 2007 | Longe O A | Cerebral Cortex | 12 | 1 | Stems (Nouns + Verbs) vs. Baseline | Talairach | -40 -24 42  -56 -22 37  -57 13 16  -44 22 28  6 -63 -15  20 -56 -28  -34 -46 -23  -42 -49 -18 |
| 11110102 | 2007 | Longe O A | Cerebral Cortex | 12 | 2 | Inflections (Nouns + Verbs) vs. Baseline | Talairach | -42 -47 -16  -44 -63 -19  -55 -19 38  -38 -23 42  -48 -33 46  -48 24 17  -40 7 25  -48 11 12  -50 27 4  -36 33 0  -32 30 -12  4 -49 -6  6 -71 -20  16 -55 -16 |
| 11110102 | 2007 | Longe O A | Cerebral Cortex | 12 | 3 | Stems + Inflections vs. Baseline | Talairach | -55 -19 38  -40 -23 42  -46 -25 47  4 -49 -4  -42 -49 -18  -34 -46 -23  -46 -61 -17  -4 12 56  -40 9 27  -28 -12 -4  -48 12 12  -50 22 19  -57 12 16 |
| 11110102 | 2007 | Longe O A | Cerebral Cortex | 12 | 4 | Inflected vs. Stem | Talairach | -38 16 14  -44 7 18  -24 35 -8  -63 -39 4 |
| 11110102 | 2007 | Longe O A | Cerebral Cortex | 12 | 5 | Inflected Verbs vs. Verb Stems | Talairach | -22 19 -14  -59 -22 -3 |
| 11110102 | 2007 | Longe O A | Cerebral Cortex | 12 | 6 | Inflected Nouns vs. Noun Stems | Talairach | -16 -66 36  -14 -66 25  -26 -58 34  -49 -60 5 |
| 11110102 | 2007 | Longe O A | Cerebral Cortex | 12 | 7 | Inflected Verbs vs. Inflected Nouns | Talairach | -40 8 12  -51 -42 9 |
| 11110102 | 2007 | Longe O A | Cerebral Cortex | 12 | 8 | (Inflected Verbs vs. Verb Stems) vs. (Inflected Nouns vs. Noun Stems) | Talairach | -46 -24 -14  -22 22 -14 |
| 30203 | 2002 | Luke K | Human Brain Mapping | 7 | 4 | English Semantic - English Font | Talairach | -31 20 28  -30 62 8  -50 19 1  -55 8 32  -25 5 55  51 26 -11  50 18 21  15 59 -8  48 -2 42  -53 -45 20  -49 1 -12  43 -45 -13  -30 -65 42  33 -70 41  -15 -79 0  -24 -74 -1  29 -78 -11  -6 -15 16  -20 0 -15  -1 18 41  19 -30 -12  -39 -30 -26  6 -51 -17 |
| 12100071 | 2003 | Luo Q | Cognitive Brain Research | 10 | 1 | Semantic Jugdment > Fixation | Talairach |  |
| 12100071 | 2003 | Luo Q | Cognitive Brain Research | 10 | 2 | Analogy > Semantic Judgment | Talairach |  |
| 9110180 | 2004 | Macrae C N | Cerebral Cortex | 22 | 2 | Self Relevant > Not Self Relevant | MNI | -9 50 0 |
| 9110180 | 2004 | Macrae C N | Cerebral Cortex | 22 | 3 | Not Self Relevant > Self Relevant | MNI | 2 19 40 |
| 5040054 | 2004 | Maguire E A | NeuroImage | 12 | 1 | Facts vs. Baseline | MNI | -30 -15 -18  -51 -9 -21  -51 -66 18  -54 -51 30  12 -87 -27  -42 27 -9  0 -12 12  -45 21 30  -39 30 6 |
| 5040054 | 2004 | Maguire E A | NeuroImage | 12 | 2 | Control vs. Baseline | MNI | -42 36 -12  -48 24 24 |
| 13030023 | 2007 | Mashal N | Brain and Language | 15 | 1 | Novel Metaphors > Unrelated Words | Talairach | 37 18 8  -6 -11 13  -37 45 16  40 49 11  -35 18 7  -46 33 14  -36 34 32  49 22 25  -40 -48 43  -5 31 28  -51 -46 0  -52 9 21  52 15 10  63 -30 -1  43 -22 4 |
| 13030023 | 2007 | Mashal N | Brain and Language | 15 | 2 | Conventional Metaphors > Unrelated Words | Talairach | -50 -52 2  -32 -70 39  42 46 7  -37 44 17  -41 -50 39  40 50 10  -7 -11 14  -37 16 6  61 -31 -3  36 21 9  -35 38 32  -47 8 17  -42 36 8  52 15 10 |
| 13030023 | 2007 | Mashal N | Brain and Language | 15 | 3 | Literal Expressions > Unrelated Words | Talairach | -32 -70 39  -40 -48 41  40 51 19  -36 45 21  37 18 8  55 -40 -1  -52 -47 -1  -5 -11 6  52 15 10  -46 9 21  -44 36 8  -34 13 6 |
| 13030023 | 2007 | Mashal N | Brain and Language | 15 | 4 | Novel Metaphors > Literal Expressions | Talairach | -45 30 19  60 -50 14  40 28 13  -48 16 33  2 24 29 |
| 13030023 | 2007 | Mashal N | Brain and Language | 15 | 5 | Conventional Metaphors > Literal Expressions | Talairach | 51 -28 53  -51 -57 24  -44 29 -6 |
| 13030023 | 2007 | Mashal N | Brain and Language | 15 | 6 | Novel Metaphors > Conventional Metaphors | Talairach | 49 -29 4  47 21 20  -45 32 22 |
| 13030031 | 2009 | Mashal N | Laterality | 15 | 1 | Novel Metaphoric Sentences > Nonsensical Sentences | Talairach | -47 -59 25  -46 -6 -10  -54 -41 -1  -10 -50 29 |
| 13030031 | 2009 | Mashal N | Laterality | 15 | 2 | Novel Metaphoric Sentences > Literal Sentences | Talairach | -44 18 30 |
| 30156 | 2001 | Michael E B | Human Brain Mapping | 9 | 1 | Visual Object Relative - Baseline | Talairach | -52 -36 5  38 -27 7  -55 -13 16  38 -20 11  -42 11 26  45 15 28  -29 -70 -7  27 -70 -8  1 -70 5  -33 -62 40  31 -61 37  -23 -60 44  22 -64 48  -37 22 36  34 35 32  -38 0 45  35 -3 49  -4 -9 60 |
| 8060151 | 2007 | Murray L J | Journal of Neuroscience | 17 | 1 | Regions Active at the Target Word During Relational Trials Relative to Item Specific Trials | MNI | -49 10 24  -10 14 56  -52 35 18  -56 -56 0  -32 24 0  -28 -28 -24  46 14 24  32 21 -4  -38 -35 -21  -14 10 7  -35 -52 46  -32 14 56  -52 49 -7  49 38 32  -38 4 56  -38 -42 42  -14 7 66  -14 28 28  -7 10 28  -46 -56 -10  -49 7 56 |
| 12100074 | 2006 | Nakic M | NeuroImage | 13 | 2 | Low Frequency > High Frequency Words | Talairach |  |
| 30402 | 2003 | Neumann K | Journal of Fluency Disorders | 5 | 13 | Between-Group: PWS Before Therapy < PWNS | MNI | -52 34 -14  -40 18 18  -6 20 44 |
| 30402 | 2003 | Neumann K | Journal of Fluency Disorders | 5 | 14 | Between-Group: PWS After Therapy < PWNS | MNI | -52 34 -14  -40 18 18  -6 20 44 |
| 30402 | 2003 | Neumann K | Journal of Fluency Disorders | 5 | 15 | Between-Group: PWS at Follow-up < PWNS | MNI | -52 36 4  -52 34 -14  -40 18 18  -6 20 44 |
| 30402 | 2003 | Neumann K | Journal of Fluency Disorders | 5 | 16 | Between-Group: PWS Over All Assessment Times < PWNS | MNI | -52 34 -14  -36 18 18 |
| 30402 | 2003 | Neumann K | Journal of Fluency Disorders | 5 | 17 | Between-Group: PWS Over All Assessment Times > PWNS | MNI | -46 12 44  -52 14 36  -48 22 6  -48 -64 -20 |
| 13070059 | 2011 | Newman S D | Brain Research | 15 | 3 | Word Easy vs. Fixation | MNI | -2 10 46  -18 -10 2  24 -28 -2  12 -76 -14  -12 -90 -4  34 -50 46  34 -54 -28  -44 -44 50  -28 -64 50  2 -70 52  -34 -52 -28  -24 -30 8  -46 2 32  -32 2 56 |
| 13070059 | 2011 | Newman S D | Brain Research | 15 | 4 | Word Hard vs. Fixation | MNI | -4 10 48  -32 2 54  -46 -42 46  -32 -58 42  -36 -56 -16  34 -54 50  32 -56 -28  12 -74 -16  -54 12 26  -14 -90 -4  30 18 0  -16 2 2  4 -72 52  -28 18 0  12 -8 -2  8 -48 -12  26 -2 58 |
| 13070059 | 2011 | Newman S D | Brain Research | 15 | 5 | Word > Number | MNI | -4 6 58  -30 30 2  -36 10 32  24 -68 -6  -14 -76 -10  6 -86 2  2 -52 -28  8 -8 -6  46 -32 2  22 10 8 |
| 13070059 | 2011 | Newman S D | Brain Research | 15 | 7 | Hard > Easy | MNI | -20 6 64  -30 2 66  -4 12 52  -32 -52 36  -46 -50 44  -52 -46 50  30 10 62  20 6 60  24 4 68  -10 -64 48  -30 50 8  -36 58 12  -2 -30 -22  38 32 46  46 38 30  30 -54 -30  10 -70 -26 |
| 13070059 | 2011 | Newman S D | Brain Research | 15 | 8 | Interaction | MNI | -40 -68 50 |
| 13070059 | 2011 | Newman S D | Brain Research | 15 | 11 | Word Easy Correlation With Reading Span | MNI | 24 -76 6  8 52 52  -24 -98 0  0 0 72  -48 8 30  26 -100 2 |
| 13070059 | 2011 | Newman S D | Brain Research | 15 | 12 | Word Hard Correlation With Reading Span | MNI | 24 -78 4  40 8 24  44 -30 46 |
| 5040055 | 2004 | Noppeney U | NeuroImage | 15 | 1 | Abstract Concepts > Sounds, Visual Attributes, and Hand Movements | MNI | -54 21 -6  -51 18 -27  -51 9 -24  -60 -42 -6 |
| 5040055 | 2004 | Noppeney U | NeuroImage | 15 | 2 | Effect of Task Difficulty | MNI | -30 -18 -6  -6 24 48  -9 -6 3  -42 9 27  0 -57 -45 |
| 9110182 | 2005 | Ochsner K N | NeuroImage | 17 | 1 | Self > Syllable | MNI | -6 32 30 |
| 9110182 | 2005 | Ochsner K N | NeuroImage | 17 | 2 | Other > Syllable | MNI | 0 56 -4  -2 56 20  -8 40 34 |
| 9110182 | 2005 | Ochsner K N | NeuroImage | 17 | 3 | Positive > Syllable | MNI | 2 52 -10  -8 52 16  2 48 4  0 -56 30 |
| 9110182 | 2005 | Ochsner K N | NeuroImage | 16 | 4 | Appraisals > Curved Lines | MNI | -12 48 36  -10 52 4  -12 28 52  18 38 44  -4 12 -16  -38 10 48  0 -54 24  -8 -52 28  -8 -64 14  -48 -68 22  -60 -56 20  -56 -48 -4  -66 -38 2  -60 -6 -18  -52 -8 -20  -62 -24 -10  -48 4 -36  -42 20 -30  52 -62 22  56 -70 26  60 -62 20  62 -10 -18  12 -76 -10 |
| 30164 | 1999 | Poldrack R A | NeuroImage | 8 | 1 | Semantic > Case | Talairach | -46 20 -3  -1 20 42  -49 8 26  38 50 15  -1 35 47  -2 8 45  34 20 0  7 60 -18  52 8 26  -46 50 1  -35 35 -1  43 35 13  12 8 12  -12 0 18  -23 60 -7  -25 50 -16  -5 50 33  -10 16 11  -39 0 47 |
| 30164 | 1999 | Poldrack R A | NeuroImage | 8 | 5 | Semantic > Phonological | Talairach | -7 50 31  -4 35 48  -53 16 25  4 20 56  -37 28 -9  -42 40 -8 |
| 30164 | 1999 | Poldrack R A | NeuroImage | 8 | 9 | Semantic > Phonological (Double Subtraction) | Talairach | -44 20 -1  49 16 30  47 28 -9  39 40 -10  15 55 -14  -47 8 21  10 8 11  -24 50 -16  34 55 7  -5 55 20  -35 35 -2  -22 60 -8  -12 0 23  -12 16 12  -9 40 10  -8 28 12 |
| 30164 | 1999 | Poldrack R A | NeuroImage | 8 | 11 | Semantic > Pseudoword Phonological (Double Subtraction) | Talairach | -10 28 54  -7 35 34  -55 20 17  -6 16 54  -13 55 24  17 40 42  -43 50 0  -33 35 -9  -39 0 55  49 20 -7  -44 8 26  41 35 -8  -12 16 13  52 8 39  3 16 4  15 60 24  32 55 5  -34 8 -6 |
| 6120186 | 2001 | Poldrack R A | Brain | 16 | 1 | New MR vs. Plain Text, Mirror Reading I, Pre-Training | MNI | -24 -69 45  -45 6 24  39 -72 -21  -45 -54 -18  36 -42 42  -12 60 -12  21 -30 -6  30 -51 -21  3 -27 -18  12 60 -9  24 9 3  12 0 3  30 30 -3 |
| 6120186 | 2001 | Poldrack R A | Brain | 16 | 2 | New MR vs. Plain Text, Mirror Reading I, Post-Training | MNI | -48 0 36  -24 -63 45  42 -78 -15  -6 -27 -21  48 9 27  -15 -51 3  24 -27 -3 |
| 6120186 | 2001 | Poldrack R A | Brain | 16 | 3 | Learning-Related Increase | MNI | 24 -60 -24  -42 3 39  -54 -60 -15  -24 -66 -9  -9 18 42  15 -78 -9  9 -15 15  15 12 42  -18 0 45  12 -51 -3 |
| 6120186 | 2001 | Poldrack R A | Brain | 16 | 4 | Learning-Related Decrease | MNI | -15 -63 -21  -27 -12 -18 |
| 6120186 | 2001 | Poldrack R A | Brain | 16 | 5 | Short-Term Priming, Pre-Training | MNI | -21 36 -9  -39 -42 -9  33 -42 -6  -21 -78 -9  24 -48 18 |
| 6120186 | 2001 | Poldrack R A | Brain | 16 | 6 | Short-Term Priming, Post-Training | MNI | -45 3 24  -12 -48 6  3 48 -15  24 -84 0  -30 -6 45  15 -72 -9  -21 -75 -18  -18 -75 42  -24 -81 18  -54 30 18  -42 3 27  -24 -63 45  -6 15 42  27 -66 42  30 27 0  48 9 27  -3 -27 -18 |
| 6120186 | 2001 | Poldrack R A | Brain | 16 | 7 | Pre-Training, Mirror Reading II: SB > MR | MNI | 48 27 -3 |
| 6120186 | 2001 | Poldrack R A | Brain | 16 | 8 | Post-Training, Mirror Reading II: SB > MR | MNI | -6 -81 -18 |
| 6120186 | 2001 | Poldrack R A | Brain | 16 | 9 | Pre-Training, Mirror Reading II: MR > SB | MNI | 9 -48 -6  -9 -45 -6  3 21 9  27 -24 -3 |
| 6120186 | 2001 | Poldrack R A | Brain | 16 | 10 | Post-Training, Mirror Reading II: MR > SB | MNI | -21 27 42  -42 -30 -21  -12 -48 -27  -15 45 6  -45 -24 45  12 -45 0  -12 -39 45  -30 36 -9  45 -30 24  12 48 0  33 -54 -18  -12 -51 3 |
| 6120186 | 2001 | Poldrack R A | Brain | 16 | 11 | Pre-Training, Mirror Reading II: IR > MR | MNI | 42 -66 -21  48 24 0  30 -45 -21  -33 -72 -18 |
| 6120186 | 2001 | Poldrack R A | Brain | 16 | 12 | Post-Training, Mirror Reading II: IR > MR | MNI | 42 -66 -21  39 -36 42  -39 -57 -21  24 -78 42  -9 -81 -21  18 -78 -21  0 -21 -15  -15 -78 42 |
| 6120186 | 2001 | Poldrack R A | Brain | 16 | 13 | Pre-Training, Mirror Reading II: MR > IR | MNI | 9 -45 -9  -12 -90 -3  -51 24 24 |
| 6120186 | 2001 | Poldrack R A | Brain | 16 | 14 | Post-Training, Mirror Reading II: MR > IR | MNI | -6 48 -12  9 -45 -3  -15 -48 -27  0 -57 33 |
| 6120187 | 1998 | Poldrack R A | Cerebral Cortex | 6 | 1 | Mirror Reversed Increases, Pre-Training | Talairach | 10 -72 15  -23 -79 28  24 -72 39  -24 -64 -15  19 -72 -10  -33 -72 -33  23 -72 -35  -6 -56 -6  14 -25 13  -8 -25 9  -5 -87 9 |
| 6120187 | 1998 | Poldrack R A | Cerebral Cortex | 6 | 2 | Mirror Reversed Decreases, Pre-Training | Talairach | 4 -95 19  -55 -64 16  -2 -72 32  -6 -56 59  22 -41 69  -52 -33 11  66 -33 16 |
| 6120187 | 1998 | Poldrack R A | Cerebral Cortex | 6 | 3 | Skill-Related Increases | Talairach | 0 -72 40  -3 -72 53  34 -64 -33  -43 -56 -3  66 -32 15 |
| 6120187 | 1998 | Poldrack R A | Cerebral Cortex | 6 | 4 | Skill-Related Decreases | Talairach | -4 -87 -5  -38 -95 13  -19 -95 20  -44 -87 -3  -47 -79 21  25 -95 21  45 -87 3  27 -79 -26  32 -48 -36  31 -56 49  47 -64 40  58 -41 -15  47 -56 -10  12 -33 -7 |
| 6120187 | 1998 | Poldrack R A | Cerebral Cortex | 6 | 5 | Item-Specific Increases | Talairach | -42 -79 32  -2 -72 27  50 -72 26  1 -64 40  8 -56 68  -67 -33 -10  66 -25 -9 |
| 6120187 | 1998 | Poldrack R A | Cerebral Cortex | 6 | 6 | Item-Specific Decreases | Talairach | -23 -95 2  18 -79 -4  -33 -87 23  -16 -87 12  -39 -56 53  19 -25 51  30 -64 33  -38 -48 38  1 -64 -20  25 -72 -10  25 -64 -23  41 -64 -28  -24 -64 -24  -31 -56 -8  -28 -56 0  -10 -25 12 |
| 30410 | 2003 | Preibisch C | NeuroImage | 16 | 4 | PDS > Controls, Fixed effects further masked inclusive by fixed effects difference Semantic Decision | MNI | 36 18 -16 |
| 30410 | 2003 | Preibisch C | NeuroImage | 16 | 7 | Controls > PDS, Fixed effects further masked inclusive by fixed effects difference Semantic Decision | MNI | -46 -2 32 |
| 8060155 | 2005 | Prince S E | Journal of Neuroscience | 14 | 3 | Semantic, (Encoding + Retrieval) > Perceptual, (Encoding + Retrieval) | Talairach | -46 26 2  -23 28 -17  -34 -22 5  -15 26 -1  -23 1 10  23 10 -13  -19 -1 -13  -19 -56 -22 |
| 60100159 | 2005 | Ragland J D | American Journal of Psychiatry | 14 | 1 | Deep Word Encoding - Shallow Word Encoding, Normals | Talairach | -40 30 -12  -20 46 0  -40 14 16 |
| 60100159 | 2005 | Ragland J D | American Journal of Psychiatry | 14 | 3 | Deep Word Encoding - Shallow Word Encoding, Schizophrenics > Normals | Talairach | -8 -30 4  -32 -42 -4  -32 -58 -4 |
| 60100159 | 2005 | Ragland J D | American Journal of Psychiatry | 14 | 11 | Deep Encoding, Normals | Talairach | 16 -86 0  -44 46 4  -24 -58 40  -8 -18 -4  52 10 32  4 10 52  -28 18 0  -12 2 4 |
| 60100159 | 2005 | Ragland J D | American Journal of Psychiatry | 14 | 13 | Deep Encoding, Normals - Schizophrenics | Talairach | 16 -86 0  -8 10 8  8 34 48  24 54 20  -24 50 -4  36 -62 32  4 -38 28  40 30 12  48 -50 36 |
| 60100159 | 2005 | Ragland J D | American Journal of Psychiatry | 14 | 14 | Deep Encoding, Schizophrenics - Normals | Talairach | -4 -10 4  -44 -6 40 |
| 13030032 | 2004 | Rapp A | Cognitive Brain Research | 15 | 1 | Metaphoric Sentences > Baseline | Talairach | -24 -91 -6  39 -90 2  -50 -34 5  -50 37 -12  -56 -19 37  -21 23 -14  -53 16 32  6 16 38  -9 -29 -4  -15 -6 0  -36 -47 52 |
| 13030032 | 2004 | Rapp A | Cognitive Brain Research | 15 | 2 | Literal Sentences > Baseline | Talairach | -21 -88 -6  39 -90 2  -50 -35 5  36 23 -6  -42 -6 50  -27 25 -21  -12 16 38  -9 -26 -4  15 3 -3  -33 -42 41  -53 17 -11  53 17 -16 |
| 13030032 | 2004 | Rapp A | Cognitive Brain Research | 15 | 3 | Metaphoric Sentences > Literal Sentences | Talairach | -39 35 1  -45 -7 -30  -53 -58 -2 |
| 7060156 | 2006 | Ries M L | NeuroImage | 14 | 2 | Self-Appraisal vs. Semantic Decision, Normals | MNI | 8 -62 14  -8 -54 28  8 -58 24  10 58 24  -12 58 8  -10 48 42 |
| 7060156 | 2006 | Ries M L | NeuroImage | 14 | 3 | Cognitive Conjunction, Normals | MNI | 8 -60 24  4 -66 22 |
| 30224 | 2002 | Rossell S L | Brain and Language | 6 | 1 | Areas of Activation During Lexical Visual Field Performance for Males | Talairach | -35 -64 -7  -38 -61 -13  -40 0 26  -23 -92 -7  -40 6 37  49 5 37  32 -58 42 |
| 30224 | 2002 | Rossell S L | Brain and Language | 6 | 2 | Areas of Activation During Lexical Visual Field Performance for Females | Talairach | -23 -89 -13  -38 -50 -13  32 -44 -13  -35 0 31  -46 25 20  43 11 31  -43 -61 -7  61 -19 -2 |
| 30224 | 2002 | Rossell S L | Brain and Language | 6 | 3 | Greater Activation in Males | Talairach | -43 -53 -7  -46 -72 -7  32 -58 42 |
| 30224 | 2002 | Rossell S L | Brain and Language | 6 | 4 | Greater Activation in Females | Talairach | 49 19 26  40 8 31  64 -14 -2 |
| 30225 | 2001 | Rossell S L | Neuropsychologia | 12 | 1 | Related and Unrelated Prime-Target Pairs During Semantic Priming, Short SOA Tasks | Talairach | -3 47 9  -6 31 15  35 6 4  -6 -53 26  52 3 4 |
| 30225 | 2001 | Rossell S L | Neuropsychologia | 12 | 2 | Related and Unrelated Prime-Target Pairs During Semantic Priming, Long SOA Tasks | Talairach | 0 8 31  0 8 37  35 -6 9  32 -8 4  -3 -47 31  -20 -50 9  38 -28 4  -38 -8 -13  52 0 -13  49 0 -18  -38 -36 -7  20 -33 -7  -17 8 -7  23 -8 -2  55 -17 15 |
| 30225 | 2001 | Rossell S L | Neuropsychologia | 12 | 3 | Lexical Decision Only, Short SOA Tasks | Talairach | 43 25 20  49 17 26  -43 22 15  -40 14 31  -43 -67 -7  20 -89 -13  -38 -64 -13  -45 -56 -18  -14 -67 4  -19 -64 -2  23 -81 -7 |
| 30225 | 2001 | Rossell S L | Neuropsychologia | 12 | 4 | Lexical Decision Only, Long SOA Tasks | Talairach | 49 14 31  49 11 26  -32 6 31  23 -81 -7  -35 -67 -7  -40 -64 -13  -12 -83 -2  23 -81 -7 |
| 12100077 | 2005 | Sabsevitz D S | NeuroImage | 28 | 1 | Concrete > Abstract | Talairach |  |
| 12100077 | 2005 | Sabsevitz D S | NeuroImage | 28 | 2 | Abstract > Concrete | Talairach |  |
| 13060045 | 2009 | Schmidt G | Brain and Cognition | 10 | 1 | All Sentences (Literal, Easy-Familiar, Easy-Unfamiliar, Difficult-Unfamiliar) > Non-Word Sentences | Talairach | -34 18 13  27 -3 24  -34 -10 -13 |
| 13060045 | 2009 | Schmidt G | Brain and Cognition | 10 | 2 | Metaphors (Easy-Familiar, Easy-Unfamiliar, Difficult-Unfamiliar) > Literal Sentences | Talairach | -64 -8 27  36 -4 8  -57 21 -9  -42 -38 27  13 -51 40  -18 -72 -1 |
| 13060045 | 2009 | Schmidt G | Brain and Cognition | 10 | 3 | Easy-Familiar Metaphors > Literal Sentences | Talairach | 6 -65 34  -64 -9 24  -13 -78 22  15 -85 22  -24 -65 13  -31 -80 7  -27 -73 1  60 28 -4  -29 -61 -4  -15 -32 -10  14 12 -26 |
| 13060045 | 2009 | Schmidt G | Brain and Cognition | 10 | 4 | Familiar Metaphors (Easy-Familiar) > Unfamiliar Metaphors (Easy-Unfamiliar) | Talairach | 49 18 38  -29 -99 7  -24 -89 2  25 -52 1 |
| 13060045 | 2009 | Schmidt G | Brain and Cognition | 10 | 5 | Unfamiliar Metaphors (Easy-Unfamiliar) > Familiar Metaphors (Easy-Familiar) | Talairach | -12 -11 30  -29 -36 -2 |
| 13060045 | 2009 | Schmidt G | Brain and Cognition | 10 | 6 | Easy Metaphors (Easy-Unfamiliar) > Difficult Metaphors (Difficult-Unfamiliar) | Talairach | -24 -54 49  -46 37 22  1 20 18  -5 30 17  -10 40 3  -15 19 5  17 34 1  -28 0 -6 |
| 13060045 | 2009 | Schmidt G | Brain and Cognition | 10 | 7 | Difficult Metaphors (Difficult-Unfamiliar) > Easy Metaphors (Easy-Unfamiliar) | Talairach | -33 -73 46  -65 11 34 |
| 9110183 | 2004 | Schmitz T W | NeuroImage | 19 | 1 | Self Evaluation > Semantic Positivity Evaluation | MNI | 6 56 4  2 -60 16  2 -24 2  -4 18 -10 |
| 9110183 | 2004 | Schmitz T W | NeuroImage | 19 | 2 | Significant Other Evaluation > Semantic Positivity Evaluation | MNI | -4 58 4  2 -58 16  -2 -2 4 |
| 9110183 | 2004 | Schmitz T W | NeuroImage | 19 | 3 | Self Evaluation > Significant Other Evaluation | MNI | 26 52 16  -28 46 16  30 -40 -14 |
| 7070188 | 2008 | Seghier M L | Human Brain Mapping | 50 | 1 | Semantic Categorization vs. Perceptual Categorization | MNI | -50 14 -13  -48 27 15  -45 15 22  -50 -38 3  -30 -65 42  -48 5 47  -3 20 46  42 20 -9  39 54 22  50 -32 2 |
| 7070190 | 2007 | Soderlund H | NeuroImage | 12 | 1 | Word Encoding, Alcohol = Placebo | Talairach | -44 26 3  -9 43 42  -5 19 52  -34 10 46 |
| 7070190 | 2007 | Soderlund H | NeuroImage | 12 | 2 | Word Encoding, Alcohol NOT= Placebo | Talairach | -48 23 18  48 -34 3  37 42 -4  3 14 60  -35 -52 -22  -1 -62 56  -25 -90 -6  -19 -8 29  -42 -32 30  23 -42 -24  12 -91 34  -44 22 -2 |
| 7070190 | 2007 | Soderlund H | NeuroImage | 12 | 3 | Phrase-Word Pair Encoding, Alcohol = Placebo | Talairach | -40 26 5  -36 -72 -13  -39 0 41  -5 14 52 |
| 7070190 | 2007 | Soderlund H | NeuroImage | 12 | 4 | Phrase-Word Pair Encoding, Alcohol NOT= Placebo | Talairach | -42 -50 58  8 -49 45  41 -40 25 |
| 9090154 | 2003 | Sonty S | Annals of Neurology | 14 | 3 | SYN > STRINGS, Normals | MNI | -45 39 -12  -6 21 39  -39 -60 24  -42 -69 30  42 33 -15  45 -9 15  -45 -27 0  51 -24 -3 |
| 9090154 | 2003 | Sonty S | Annals of Neurology | 14 | 6 | PPA Patients > Normals, SYN | MNI | -54 3 15  51 3 21  27 -69 -18  -27 -60 54 |
| 13030024 | 2007 | Stringaris A K | Brain and Language | 11 | 1 | Metaphoric > Literal | Talairach | -29 -59 -29  14 -81 -13  -7 -15 4  -43 29 -2  32 -67 15  -21 -81 20  25 -66 25  -51 -11 36  -29 -59 42 |
| 13030024 | 2007 | Stringaris A K | Brain and Language | 11 | 2 | Metaphoric > Non-Meaningful | Talairach | -7 -15 4 |
| 13030024 | 2007 | Stringaris A K | Brain and Language | 11 | 3 | Non-Meaningful > Metaphoric | Talairach | -25 -85 -13  -40 -70 -13  43 -59 -18  -32 26 -7  14 -52 9  -11 -77 9  47 4 20  -43 18 20  -14 -74 31  47 -63 -7  11 -63 20  -26 -44 37  32 -52 42  -4 -56 37  11 -33 48  0 -7 48  -40 -26 42 |
| 13030024 | 2007 | Stringaris A K | Brain and Language | 11 | 4 | Literal > Metaphoric | Talairach | -32 -63 -18  -22 -78 -12  0 56 -13  47 -63 -7  -29 -78 15  7 -41 42  40 -11 42 |
| 13030024 | 2007 | Stringaris A K | Brain and Language | 11 | 5 | Literal > Non-Meaningful | Talairach | -51 -7 -7  -22 -74 15  47 15 20  36 -7 42 |
| 13030024 | 2007 | Stringaris A K | Brain and Language | 11 | 6 | Non-Meaningful > Literal | Talairach | -18 -56 -40  40 -59 -18  -32 18 -2  32 48 -2  -47 -52 4  -22 -78 9  14 -67 20  54 -4 20  -29 -56 37  29 -59 42  -4 -7 48  0 -56 53  -40 -30 48 |
| 13030025 | 2006 | Stringaris A K | NeuroImage | 12 | 1 | Literal Followed by Irrelevant (IRL) > Metaphoric Followed by Irrelevant (IRM) | Talairach | -25 -78 15  22 -22 9  47 -59 -7  29 -70 26  -32 -56 37  -11 -59 42  0 -37 48  -29 -30 48 |
| 13030025 | 2006 | Stringaris A K | NeuroImage | 12 | 2 | Metaphoric Followed by Irrelevant (IRM) > Literal Followed by Irrelevant (IRL) | Talairach | -29 -48 -29  -11 -70 -18  36 26 -7  -32 -52 42 |
| 13030025 | 2006 | Stringaris A K | NeuroImage | 12 | 3 | Literal Followed by Relevant (RL) > Metaphoric Followed by Relevant (RM) | Talairach | 51 -4 -29  40 -67 -7  29 26 -2  -51 -11 26 |
| 13030025 | 2006 | Stringaris A K | NeuroImage | 12 | 4 | Metaphoric Followed by Relevant (RM) > Literal Followed by Relevant (RL) | Talairach | -3.6 -48 -35  -32 -74 -7  43 30 -7  -40 4 37 |
| 7110312 | 2007 | Suh S | Brain Research | 16 | 2 | Embedded English vs. Rest | MNI | -44 24 16  -6 24 40  -34 -60 46  -24 -94 -15  36 -58 52 |
| 7110312 | 2007 | Suh S | Brain Research | 16 | 4 | Conjoined English vs. Rest | MNI | -2 10 60  34 -62 46  12 -90 -10 |
| 7110312 | 2007 | Suh S | Brain Research | 16 | 6 | Embedded Korean vs. Embedded English | MNI | -26 -80 2  8 -85 14  50 -34 46  -60 0 30  -16 -76 6  -34 45 8  4 30 48  -40 24 44  -42 23 -10  62 -10 -20 |
| 7110312 | 2007 | Suh S | Brain Research | 16 | 7 | Embedded English vs. Embedded Korean | MNI | 30 -80 30  -18 -34 50  -18 -70 24 |
| 7110312 | 2007 | Suh S | Brain Research | 16 | 8 | Conjoined Korean vs. Conjoined English | MNI | -38 7 10  -30 -68 -2  -32 20 -18 |
| 7110334 | 2007 | Thompson C K | Journal of Cognitive Neuroscience | 17 | 1 | Pseudowords vs. Words | Talairach | -45 10 19  59 7 30  -39 32 1  -45 2 30  45 0 8  -53 -19 31  53 -21 51  42 -21 51  48 -26 57  -36 -36 38  -45 -30 37  -53 5 -10  -48 -41 5  -56 -49 8  -50 -69 9  -9 14 41  3 17 41  9 22 35  12 21 21  -39 -64 -4  -48 -68 -12  48 -62 -12  -12 -56 -10  -18 -59 -15  -33 -42 -21 |
| 7110334 | 2007 | Thompson C K | Journal of Cognitive Neuroscience | 17 | 2 | Verbs vs. Fixation | Talairach | -56 7 30  -59 -16 37  -30 18 5  -42 -2 8  56 2 39  62 4 25  -53 -7 42  -6 5 47  6 8 47  39 -3 50  53 -26 7  65 -23 15  -39 -61 9  -45 -33 46  39 -41 49  56 -20 18  -30 -53 52  15 -96 2  -24 -90 2  -27 -78 18  -30 -73 -11  -39 -71 -14  33 -82 -9  45 -74 -14  6 9 11  6 15 2  -3 -24 -4  12 -17 9 |
| 7110334 | 2007 | Thompson C K | Journal of Cognitive Neuroscience | 17 | 3 | Nouns vs. Fixation | Talairach | 33 20 2  42 24 21  33 23 -9  45 -15 45  -30 15 5  -42 -5 9  -48 3 5  -50 -7 39  -56 4 27  36 19 27  42 -12 53  -6 5 47  36 19 27  53 -34 16  -42 -36 40  -39 -41 52  -33 -47 52  30 -65 39  -27 -68 31  -30 -53 55  -9 7 30  -39 -79 -11  27 -90 -1  -21 -93 2  18 -96 5  -36 -48 -20  30 -42 -18  -18 0 -5  -27 3 -5  -6 -24 -9  12 -14 3 |
| 60100164 | 2005 | Tieleman A | NeuroImage | 22 | 1 | Semantic Self-Paced vs. Perceptual Self-Paced | Talairach | -50 26 10  -36 32 -18  -52 18 21  -44 16 26  -2 18 51  2 32 54  -2 32 54  -2 23 41  34 29 -12  32 34 -15  -48 9 -14  -36 20 14  -50 7 13  -50 -41 2  42 15 -18  -26 -16 -16  -24 -24 -12  -26 -30 -17  -18 -13 -16  -36 -44 -16  18 -14 -12  -10 -89 10  -12 85 14  -8 -78 4  -36 -44 -17  -4 -70 16  12 -87 8  12 -87 14  14 -66 11  -8 -60 6 |
| 60100164 | 2005 | Tieleman A | NeuroImage | 22 | 2 | Semantic Fixed-Paced vs. Perceptual Fixed-Paced | Talairach | -46 17 19  -38 23 -8  -52 19 23  -48 0 42  -2 13 58  4 20 42  -8 16 49  34 23 42  46 22 -6  -46 17 -13  -38 12 12  -54 -36 2  -12 -91 5  -22 -88 -7  -20 -89 -6  -40 -49 -13  4 -49 -13  12 -90 17  8 -75 -25  34 -54 -28 |
| 60100164 | 2005 | Tieleman A | NeuroImage | 22 | 3 | Self-Paced > Fixed-Paced, Semantic vs. Perceptual | Talairach | -24 -26 -11  -20 -16 -19  -32 -20 -19  -48 -68 24  -40 11 -21  18 -14 -9  -2 -75 24  -16 -46 4  12 -83 6  20 -56 8 |
| 60100164 | 2005 | Tieleman A | NeuroImage | 22 | 4 | Greater Task-Related Deactivation During Fixed-Paced Compared to Self-Paced, Semantic | Talairach | -24 -26 -22  20 -20 -16  -54 -62 22  -50 -69 24  -48 -69 22  -20 32 46  -10 -69 22  16 -65 22  -10 -61 16  16 -50 4 |
| 11110112 | 2008 | Tyler L K | Journal of Cognitive Neuroscience | 15 | 1 | Stems (Nouns + Verbs) vs. Baseline | MNI | -48 22 28  20 -52 -32  -30 -8 -4 |
| 11110112 | 2008 | Tyler L K | Journal of Cognitive Neuroscience | 15 | 2 | Phrases (Nouns + Verbs) vs. Baseline | MNI | -52 20 28  -28 -2 -6  -52 -44 -4 |
| 11110112 | 2008 | Tyler L K | Journal of Cognitive Neuroscience | 15 | 3 | Noun Phrases vs. Baseline | MNI | -50 30 12  -54 -44 -2  -32 4 -4 |
| 11110112 | 2008 | Tyler L K | Journal of Cognitive Neuroscience | 15 | 4 | Verb Phrases vs. Baseline | MNI | -52 20 28  -30 -6 -6  -52 -42 -4  18 -54 -28 |
| 11110112 | 2008 | Tyler L K | Journal of Cognitive Neuroscience | 15 | 5 | [Phrases (Nouns + Verbs) - Baseline] vs. [Stems (Nouns + Verbs) - Baseline] | MNI | -46 -36 -6  32 -82 4 |
| 11110112 | 2008 | Tyler L K | Journal of Cognitive Neuroscience | 15 | 6 | [Noun Phrases- Baseline] vs. [Stems (Nouns + Verbs) - Baseline] | MNI | -50 -32 -6 |
| 11110112 | 2008 | Tyler L K | Journal of Cognitive Neuroscience | 15 | 7 | [Verb Phrases - Baseline] vs. [Stems (Nouns + Verbs) - Baseline] | MNI | 30 -88 6  -46 -36 -6  -52 18 28  -12 -48 40  -2 -76 22 |
| 11110112 | 2008 | Tyler L K | Journal of Cognitive Neuroscience | 15 | 8 | Verb Phrases vs. Noun Phrases | MNI | -52 -58 6 |
| 11110113 | 2003 | Tyler L K | NeuroImage | 12 | 1 | Tools vs. Letter Strings | MNI | -46 12 32  -50 20 12  -48 28 -10  -30 -34 -26  -20 -14 -14  -42 -42 -26  4 24 36  0 14 48  -6 28 54  0 -80 8  16 -82 10  10 -58 -2 |
| 11110113 | 2003 | Tyler L K | NeuroImage | 12 | 2 | Animals vs. Letter Strings | MNI | -24 -12 -18  -42 -46 -28  -48 -40 -12  -28 24 -8  -30 28 -18  -44 18 4  -8 -20 20  -6 4 24  -4 -28 16  10 -72 -34  8 -64 -26  34 28 -10  42 18 6  18 26 24  16 -26 -22  10 -20 -20  38 -28 -22  4 20 44 |
| 11110113 | 2003 | Tyler L K | NeuroImage | 12 | 3 | Tool Actions vs. Letter Strings | MNI | -50 22 10  -32 22 -6  -46 32 -10  6 22 34  -4 14 46  -4 28 44  -44 -40 -24  -20 -12 -24  -18 -10 -14  -24 -58 2  -10 -78 6  -14 -66 2  36 24 -8  44 0 -14  46 20 6  10 -76 -34  6 -60 -22  10 -50 -16 |
| 11110113 | 2003 | Tyler L K | NeuroImage | 12 | 4 | Biological Actions vs. Letter Strings | MNI | -46 30 -12  -32 32 -18  -50 18 -6  -14 -36 -2  -8 -30 -2  -16 -48 0  -26 -16 -12  -34 -16 -26  -26 -22 -18  -54 -30 -6  -44 -42 -16  -52 -46 -8  -6 -68 -34  6 -36 -26  6 -62 -24  2 18 40  -4 32 40 |
| 11110113 | 2003 | Tyler L K | NeuroImage | 12 | 5 | Tool Actions vs. Tools | MNI | -50 16 14  -44 32 -14  -38 12 6 |
| 11110113 | 2003 | Tyler L K | NeuroImage | 12 | 6 | Biological Actions vs. Animals | MNI | -50 20 10  -36 28 -2  -42 24 18  -60 -50 2  -52 -32 -4 |
| 12070059 | 2004 | Tyler L K | Neuropsychologia | 12 | 1 | Words (Nouns + Verbs) - Letter Strings | MNI | -20 -14 -14  -34 -26 -26  -42 -46 -28  38 -20 44  26 -22 42  -50 22 10  -28 24 -8  -50 18 -6  4 22 34  -2 14 48  -4 28 46  4 -62 -26  10 -76 -34  -8 -18 20  -18 -4 20  -2 -28 12  34 26 -12  12 -22 -22 |
| 12070059 | 2004 | Tyler L K | Neuropsychologia | 12 | 2 | Verb - Nouns | MNI | -50 16 12  -38 22 0  -46 22 6 |
| 13030026 | 2006 | Uchiyama H | Brain Research | 20 | 2 | Sarcasm Detection: Sarcastic plus Non-Sarcastic Responses minus Unconnected | MNI | -6 16 58  -6 44 42  -8 60 28  -60 22 12  -56 24 -6  -46 18 -10  -52 8 -30  -56 -10 -12  -56 -28 -2  28 -82 -34 |
| 30181 | 2002 | Vaidya C J | Neuropsychologia | 8 | 1 | Encoding - Pictures vs. Words | MNI | -17 -66 -8  25 -54 -7  37 -85 17 |
| 13060046 | 2007 | Vorhold V | Neuropsychologia | 13 | 1 | Risk Rating > Letter Decision | MNI | -6 54 33  -42 33 -21  33 87 -42 |
| 10080210 | 2001 | Wagner A D | Neuron | 14 | 1 | 4 Target > 2 Target | MNI | -36 21 27  -39 6 24  -45 27 9  3 30 36  3 15 42  -51 21 -12 |
| 10080210 | 2001 | Wagner A D | Neuron | 14 | 2 | Weak > Strong, Associative Strength | MNI | -45 27 -12  -51 18 27  -51 21 -3  6 18 39  9 27 36  0 9 57  45 21 6  30 24 -6  39 27 -9  54 24 27  45 9 27  51 9 33 |
| 10080210 | 2001 | Wagner A D | Neuron | 14 | 3 | Weak/2 Target > Strong/4 Target | MNI | 51 21 27  54 12 21  54 12 36  -48 27 -12  -42 33 -12  -9 6 48  -48 30 27  -54 21 24  -57 12 30  6 6 54 |
| 10080210 | 2001 | Wagner A D | Neuron | 14 | 4 | 4 Target > 2 Target, Weak Association | MNI | -36 21 27  -45 15 16  -39 9 27  -45 30 0  -45 27 9 |
| 10080210 | 2001 | Wagner A D | Neuron | 14 | 5 | 4 Target > 2 Target, Strong Association | MNI | -45 21 24  -39 21 30  -39 6 27  -51 21 -12 |
| 10080210 | 2001 | Wagner A D | Neuron | 14 | 6 | Weak > Strong, 2 Target | MNI | -36 6 30  -51 18 27  -33 15 30  -48 27 -12  -36 27 3 |
| 10080210 | 2001 | Wagner A D | Neuron | 14 | 7 | Weak > Strong, 4 Target | MNI | -51 18 24  -45 15 15  -54 27 3 |
| 7110313 | 2003 | Wartenburger I | Neuron | 11 | 3 | German Semantic Judgment, LAHP - LALP | MNI | -36 36 6  -28 44 11  16 -48 -6 |
| 7110313 | 2003 | Wartenburger I | Neuron | 11 | 4 | German Semantic Judgment, LALP - LAHP | MNI | -56 4 28  48 32 28  52 20 33 |
| 7110313 | 2003 | Wartenburger I | Neuron | 12 | 7 | German Semantic Judgment - Italian Semantic Judgment, LAHP | MNI | -40 12 -6  -48 12 0  -56 8 -6  32 20 -6 |
| 7110313 | 2003 | Wartenburger I | Neuron | 11 | 8 | German Semantic Judgment - Italian Semantic Judgment, LALP | MNI | -44 4 28  -56 4 28  -52 4 39  32 16 0  32 24 -11 |
| 30365 | 2003 | Wood J N | Journal of Cognitive Neuroscience | 20 | 1 | Social SEC vs. Control | Talairach | -12 41 42  -8 48 31  -16 30 50 |
| 30365 | 2003 | Wood J N | Journal of Cognitive Neuroscience | 20 | 2 | Nonsocial SEC vs. Control | Talairach | 0 11 -11  -8 -24 68  16 30 50 |
| 30365 | 2003 | Wood J N | Journal of Cognitive Neuroscience | 20 | 3 | Social Semantic vs. Control | Talairach | -12 34 50  -4 45 38  -48 27 -8  -28 34 -15  0 -21 49  4 -20 64 |
| 30365 | 2003 | Wood J N | Journal of Cognitive Neuroscience | 20 | 4 | Nonsocial Semantic vs. Control | Talairach | -55 20 6  -48 27 -5 |
| 30189 | 2002 | Xu B | NeuroImage | 6 | 3 | Category Only Subjects: Categorization - Control | MNI | -2 20 60  -46 24 20  -58 -34 -2  -62 -56 10  58 -32 0 |
| 30189 | 2002 | Xu B | NeuroImage | 6 | 6 | Mixed Subjects: Categorization - Control | MNI | 34 48 22  18 18 50  -50 16 28  -58 -50 -4  58 -48 2  18 -40 52  28 20 50 |
| 5040061 | 2004 | Xue G | Neuroreport | 12 | 2 | English - Baseline | MNI | -39 15 19  -45 32 7  -36 5 36  33 -6 50  -27 -53 44  -36 -33 32  -36 -50 -10  -35 -68 -12  39 -59 -12  -21 -88 -8  33 -82 -9  -6 28 26  9 33 26  33 27 12  -21 6 -3  21 3 5  -12 -14 3  15 -15 -2 |
| 5040061 | 2004 | Xue G | Neuroreport | 12 | 3 | English - Chinese | MNI | -9 30 26  -3 -40 19  -27 -50 41 |
| 10080214 | 2009 | Ye Z | NeuroImage | 19 | 1 | Effects of Plausibility in Sentence Comprehension | MNI | -6 46 32  -40 22 24  -52 32 -4  -44 -60 30 |
| 10080214 | 2009 | Ye Z | NeuroImage | 19 | 2 | Effects of Syntax in Sentence Comprehension | MNI | -12 8 60  -54 22 14  -32 30 -4  34 -88 -10 |
| 10080214 | 2009 | Ye Z | NeuroImage | 19 | 3 | Effects of Interaction in Sentence Comprehension | MNI | 22 -50 -4  -30 -92 -12  -24 -96 -8  36 -94 -6 |
| 10080214 | 2009 | Ye Z | NeuroImage | 19 | 6 | Plausibility, Stroop Congruency and Flanker Congruency | MNI | 8 34 42  -44 20 28  -36 26 -14  -32 16 8  -44 -46 46  56 26 14 |
| 10080214 | 2009 | Ye Z | NeuroImage | 19 | 7 | Plausibility, and Stroop Congruency | MNI | -8 28 42  10 32 44  -38 22 26  -46 -48 44  -58 -30 -18  46 24 28  40 36 -10  38 -52 28 |
| 10080214 | 2009 | Ye Z | NeuroImage | 19 | 8 | Plausibility vs. Stroop Congruency and Flanker Congruency | MNI | -8 56 18  -40 -60 28 |
| 7110314 | 2006 | Yokoyama S | NeuroImage | 36 | 1 | Japanese and English vs. Controls | MNI | -46 8 30  -6 8 56  -44 46 -10  -60 -38 -4  -34 -58 46  6 16 52  44 46 -18  34 -58 46  12 -80 -26 |
| 7110314 | 2006 | Yokoyama S | NeuroImage | 36 | 3 | English vs. Control | MNI | -50 26 20  -50 20 -4  -46 6 30  -48 4 50  -36 -60 48  -10 -92 -6  14 -80 -16  48 -68 -28 |
| 7110314 | 2006 | Yokoyama S | NeuroImage | 36 | 4 | English vs. Japanese | MNI | -28 -92 -8  -6 -82 -28  -32 -76 -22 |
| 7110314 | 2006 | Yokoyama S | NeuroImage | 36 | 7 | English, Active vs. Control | MNI | -50 22 -2  -50 8 46  -8 26 44  -36 0 40  -52 -40 -2  32 -74 -32  8 -82 -26  -4 -82 -24 |
| 7110314 | 2006 | Yokoyama S | NeuroImage | 36 | 8 | English, Passive vs. Control | MNI | -44 24 -6  -46 22 -18  -48 6 38  -4 6 68  -8 -88 -2  12 -84 -22 |
| 7110314 | 2006 | Yokoyama S | NeuroImage | 36 | 10 | English, Passive vs. Active | MNI | -50 20 -10  -6 -88 0 |
| 9090162 | 2009 | Yoon H W | Neuroscience Letters | 12 | 1 | Word Encoding > Fixation, Alcoholic Patients > Normals | MNI | -50 -4 -20  -28 -50 50  -2 46 -8 |
| 9090162 | 2009 | Yoon H W | Neuroscience Letters | 12 | 2 | Word Encoding > Fixation, Normals > Alcoholic Patients | MNI | -30 -52 -8  44 -58 -14  16 -74 2  -12 -76 3 |
| 12070061 | 2011 | Yu X | NeuroImage | 20 | 1 | (Nouns - Fixation) > (Verbs - Fixation) | MNI | -42 33 -18  -12 42 42  -3 36 -24  -54 3 -33  -30 -33 -21  33 -30 -21  -33 -72 39  45 -75 45 |
| 12070061 | 2011 | Yu X | NeuroImage | 20 | 2 | (Verbs - Fixation) > (Nouns - Fixation) | MNI | -51 9 6  -45 -51 9  51 -39 9  21 -63 9  24 -69 -48 |
| 12070061 | 2011 | Yu X | NeuroImage | 20 | 3 | High-Imageability Nouns > High-Imageability Verbs | MNI | -30 24 60  -45 39 -18  -33 15 -21  36 39 -18  -54 3 -36  -33 -36 -18  36 -33 -18  -33 -72 39  -6 -57 12 |
| 12070061 | 2011 | Yu X | NeuroImage | 20 | 4 | High-Imageability Verbs > High-Imageability Nouns | MNI | -51 9 6  39 18 12  12 -33 63  60 0 45  -57 -39 21  -36 -96 9  18 -90 18  24 -69 -48  -30 -54 -48 |
| 12070061 | 2011 | Yu X | NeuroImage | 20 | 5 | Low-Imageability Verbs > Low-Imageability Nouns | MNI | -60 3 36  36 0 30  -12 15 51  18 36 27  -66 -48 9  54 -36 9  30 -57 3  -9 -75 -39 |
